# Self-efficacy fluctuation and training engagement in adolescent athletes: online social feedback and the moderating role of comparison orientation

**DOI:** 10.3389/fpsyg.2026.1908710

**Published:** 2026-10-27

**Authors:** Yuanying Xu, Bairen Zhou, Zhichao Zhu, Mi Li

**Affiliations:** 1 College of Physical Education, Ningxia Normal University, Guyuan, Ningxia, China; 2 Graduate School of Sports Science, Dongshin University, Naju-si, Jeollanamdo, Republic of Korea; 3 College of Physical Education, Zaozhuang University, Zaozhuang, Shandong, China

**Keywords:** adolescent athletes, ecological momentary assessment, online social feedback, self-efficacy fluctuation, social comparison orientation, training engagement

## Abstract

**Introduction:**

This study treats adolescent athletes' self-efficacy as a dynamic process rather than a fixed quantity, asking how its fluctuations arise in online social settings and how that volatility shapes training engagement.

**Methods:**

We recruited a stratified sample of 320 youth athletes across sport discipline, competitive tier, and sex, and combined ecological momentary assessment with weekly follow-up over 8 consecutive weeks, using instruments released under open licenses and open-source software throughout. Two dispersion indices, the intraindividual standard deviation and the root mean square of successive differences, summarized fluctuation at the person level, while absolute occasion-to-occasion change indexed momentary instability at the event level. We fitted multilevel linear models, a two-level dynamic structural equation model, latent growth curve models, and multilevel structural equation models, with each path labeled by its level of analysis.

**Results:**

Momentary self-efficacy showed genuine within-person temporal dependence rather than independent scatter, and its trajectory was phase-linked to the competitive calendar and unevenly distributed across individuals. Between persons, fluctuation amplitude tracked the intensity of negatively valenced comparison rather than the sheer volume of feedback, and comparison orientation amplified that association on the negative arm alone. Within persons, negative comparison enlarged momentary instability both immediately and at a one-occasion lag, whereas positive signals steadied confidence only briefly. Amplitude was negatively associated with all three engagement facets beyond mean efficacy, most strongly for absorption, although a formal equality test separated absorption from dedication but not vigor. The direct reach of negative comparison into engagement proved facet-specific: vigor and absorption were affected, dedication was not, so the facet-by-facet hypothesis holds only in part. Sensitivity analyses adding competitive-calendar phase, splitting the series by season phase, partialling the person mean and the person-specific autoregression out of the amplitude index, entering competitive tier as a covariate, and checking participant-coded valence against automated sentiment scoring left the pattern intact.

**Discussion:**

The findings extend social cognitive theory's vicarious and persuasive sources into a temporally dense digital regime and point toward protective coaching and self-regulation strategies.

## 1 Introduction

The displacement of adolescent athletic life into networked digital spaces is no longer an emergent trend but an established condition. Where coaches once worried mainly about physical recovery and competitive scheduling, they now contend with athletes who maintain a near-continuous presence on social platforms, broadcasting performances, comparing themselves with peers, and absorbing feedback from audiences far larger than any training group (Clark et al., 2018). Timing matters. Adolescence is the developmental window when self-referential judgments are most malleable, and the online environment supplies an unusually dense stream of exactly such judgments (Steinberg, 2008). However, much of the sport-psychology literature still treats self-efficacy as though it were assessed in a quiet laboratory rather than in the noisy, reactive ecology in which young athletes form their beliefs.

Self-efficacy theory, as articulated by Bandura, holds that task-specific confidence governs effort, persistence, and resilience in the face of setback (Bandura, 1977). In sport settings, the construct predicts a broad range of training outcomes, from skill acquisition to the willingness to push through fatigue (Feltz, 1988). However, a recent meta-analysis places the confidence–performance association at a modest and moderated level rather than a uniformly strong one (Lochbaum et al., 2022). Similarly, the engagement literature has refined how training investment is conceptualized, distinguishing its components of vigor, dedication, and absorption and linking each to motivational antecedents (Schaufeli et al., 2002). Chinese scholarship has been productive here, mapping how perceived competence sustains commitment among youth in organized sport programs (Lonsdale et al., 2008). Yet these two bodies of work have largely advanced in isolation, and the connecting mechanism, how confidence beliefs translate hour by hour into the intensity of training effort, remains underspecified (Bandura, 1997).

The online dimension further complicates the picture. Studies of social media use among young people document both upward comparison that erodes confidence and supportive interaction that bolsters it, sometimes within the same session (Verduyn et al., 2017), and an experimental meta-analysis now establishes that exposure to upward comparison targets depresses self-evaluations across age and gender groups (McComb et al., 2023). Such ambivalence implies that self-efficacy in this population is not a stable trait to be measured once, but a fluctuating state responsive to the immediate social texture of a feed. Here lies the first gap we address: most existing measurement captures a single snapshot, averaging away the very volatility that may carry diagnostic information (Bolger et al., 2003). A growing recognition that within-person variation matters has not yet been matched by designs that can track it at the relevant temporal resolution.

Two further problems compound this. Time-series modeling of psychological states in athletes is rare, partly because intensive longitudinal data are costly to collect and partly because analytic tools borrowed wholesale from econometrics fit poorly to short, irregular human-sampled series (Trull and Ebner-Priemer, 2013); practical guidance for implementing such designs in sport psychology has appeared only recently (Reifsteck et al., 2021). Furthermore, researchers have seldom attempted to integrate three layers—online social behavior, the psychological state it provokes, and the behavioral training response that follows—into a single explanatory path, since researchers tend to examine one link while holding the others as background (Nezlek, 2012). The result is a fragmented evidence base that cannot tell a coach whether a particular pattern of social activity should prompt concern.

These omissions are not merely academic. If fluctuations in confidence systematically accompany reduced training investment, then ignoring them risks both wasted training load and, more seriously, the mental health of adolescents already vulnerable to the pressures of online visibility (Twenge and Campbell, 2018). Charting the regularities in this volatility is a precondition for any psychologically protective approach to youth coaching.

The study addresses these gaps in three connected ways. First, using ecological momentary assessment, we construct fluctuation indices for self-efficacy that preserve rather than smooth within-day variation. Second, we model those indices as a temporal process, fitting the dynamics rather than the mean, and we separate what we estimate within persons from what is estimated between them. Third, we trace the path by which fluctuation accompanies training engagement, joining the social, psychological, and behavioral layers into one estimable structure. A word on provenance is warranted at the outset, because the openness of this study lies in its tools rather than its records: the measurement instruments are released under open licenses and the analytic pipeline is built entirely from freely distributed software, but the momentary, weekly, and baseline data are primary records collected by the research team from recruited minors under institutional review board approval, parental consent, and participant assent (Nosek et al., 2015). The contribution is less a new theory than a way of seeing a familiar construct in motion, and of asking what its motion costs the young people who carry it.

## 2 Theoretical foundations and research hypotheses

### 2.1 Conceptual boundaries of self-efficacy fluctuation and the features of online social contexts

Self-efficacy, in Bandura's formulation, refers to a person's judgment of their capacity to organize and execute the actions required for a designated performance (Bandura, 1986). What this definition leaves open, and what matters here, is the timescale over which such judgments are read. When measured as a generalized disposition, self-efficacy behaves much like a trait: stable across weeks, resistant to single events, and predictive of broad behavioral tendencies (Sherer et al., 1982). When sampled close to the moment of action, the same construct reveals a state character, rising and falling with situational cues that the trait formulation deliberately averages out (Caprara et al., 2006). We treat this distinction not as two competing definitions but as two resolutions of one underlying belief system, and the gap between them is exactly where fluctuation lives.

A useful way to make the separation explicit is to decompose a momentary efficacy reading into a person-specific baseline and a time-varying deviation. Following the standard within-person variance partition used in intensive longitudinal psychology (Hamaker, 2012), momentary self-efficacy for an athlete *i* at occasion *t* can be written as Equation 1:


$$\begin{array}{l} SE_{i,t}=SE_{i}+\delta_{i,t},\delta_{i,t}\sim N(0,\sigma_{i}^{2}) \end{array}$$ (1)

where *SE*_*i*_ captures the trait-level set point and δ_*i,t*_ is the state fluctuation whose dispersion $\sigma_{i}^{2}$ becomes the quantity of interest. Conventional designs estimate *SE*_*i*_ and discard δ_*i,t*_ as noise.

Why should that discarded quantity be treated as a construct in its own right, rather than as measurement error around a more meaningful average? The affective dynamics literature supplies three answers that transfer cleanly to efficacy beliefs (Kuppens and Verduyn, 2017). Fluctuation can index sensitivity to environmental cues, in the sense that a belief system which moves sharply with each incoming signal is one weakly anchored in accumulated mastery experience. It can index the depletion of self-regulatory resources, since holding a judgment steady under contradictory input is effortful, and the effort is finite. It can also index a deficit in cognitive stability, the capacity to integrate discrepant evidence without wholesale revision of the self-concept. Short-term emotional variability and instability are reliably, if modestly, associated with poorer psychological adjustment (Houben et al., 2015), and the same logic gives efficacy fluctuation an adaptive significance that its mean cannot carry. Two qualifications keep the argument honest. Variability indices are partly redundant with one another and add limited incremental information once the mean and the overall variance are in the model (Dejonckheere et al., 2019). On a bounded response scale, they are arithmetically entangled with the mean itself. Both points shape how we construct and report the indices in Section 3.2 rather than undermining the construct.

For the contemporary young athlete, that surround is increasingly online, and its structural properties differ sharply from those of a face-to-face training group. Social cognitive theory identifies four sources through which efficacy beliefs are built: mastery experience, vicarious experience, social persuasion, and physiological-affective states (Bandura, 1982). Digital platforms do not introduce new sources so much as they reconfigure the delivery of two of them. Likes, comment threads, follower counts, and algorithmically surfaced peer content compress vicarious experience and social persuasion into a rapid, quantified, and continuously updating stream (Tiggemann and Slater, 2013). A single training clip posted to a feed may, within minutes, generate dozens of evaluative signals, each one a micro-instance of persuasion or comparison that the laboratory model never anticipated.

The vicarious pathway operates with unusual force online because the comparison targets are no longer the handful of teammates present at practice but a curated, often selectively impressive population of similar others (Vogel et al., 2014). When an adolescent encounters a peer of comparable age and discipline performing at a visibly higher level, the upward comparison can sharpen aspiration or, just as readily, deflate the state component δ_*i,t*_ toward a negative excursion (Collins, 1996; McComb et al., 2023). Social persuasion online is similarly distinctive: it arrives unsolicited, from weakly known or anonymous sources, and is rendered in numeric form. The persuasive weight of a coach's measured encouragement, and that of a volatile like-count, are processed through the same belief-updating channel. Yet, the latter fluctuates on a scale the former never approached (Schunk and Pajares, 2002).

Figure 1 sets out the mechanism we propose. Online interaction events, likes, comments, community comparison, and instant feedback feed the vicarious and persuasive sources, which in turn drive the momentary deviation δ_*i,t*_ around an athlete's trait baseline; the accumulated pattern of these deviations is what we later treat as the fluctuation regularity to be modeled.

**Figure 1:**
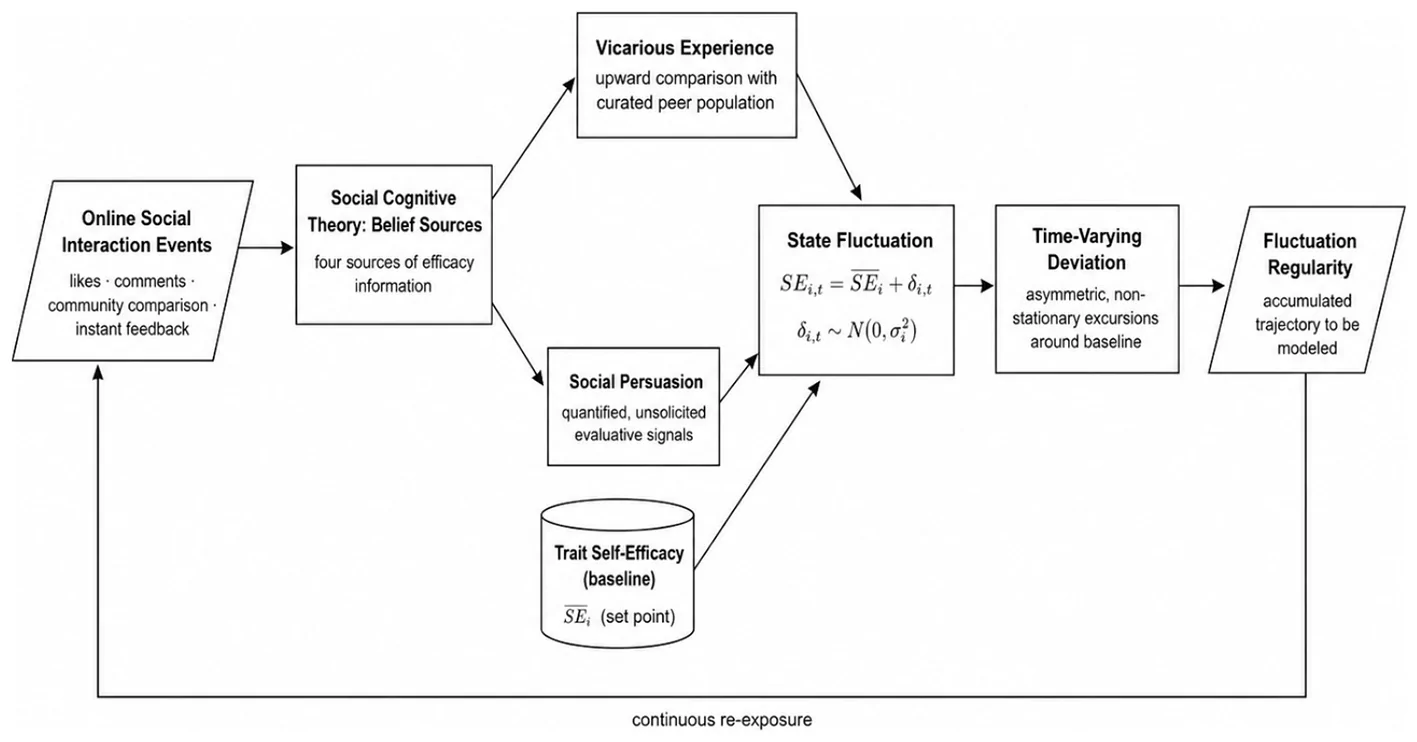
Schematic of the dynamic shaping of adolescent athletes' self-efficacy beliefs in online social contexts, tracing how discrete platform interaction events act through the vicarious-experience and social-persuasion sources of social cognitive theory to produce time-varying state deviations around a stable trait set point.

Two implications follow for handling the construct. First, because the online sources operate intermittently and asymmetrically, with negative comparison events tending to register more strongly than affirming ones among adolescents (Baumeister et al., 2001), the distribution of δ_*i,t*_ is unlikely to be symmetric or stationary, and any single-occasion measurement will misrepresent it. Second, the very feature that makes online environments developmentally hazardous, their density of evaluative feedback, is also what makes fluctuation observable at a fine temporal grain. This study therefore shifts the conceptual move from reading self-efficacy as a number and starts reading it as a trajectory whose shape is governed by an identifiable social ecology.

### 2.2 A relational framework linking online social feedback, self-efficacy, and training engagement

Training engagement, the outcome this study ultimately cares about, is best understood not as a unitary quantity of effort but as a structured psychological orientation toward training (Wong and Liem, 2022). The engagement tradition partitions it into three facets: vigor, the energy and mental resilience an athlete brings to a session; dedication, the sense of significance and pride attached to one's training; and absorption, the depth of concentrated immersion during the activity itself (Schaufeli et al., 2006; Bakker et al., 2011).

These facets differ in more than label, and the differences matter for what follows. Absorption is the most state-like of the three: it is constituted moment by moment out of attentional resources, so anything competing for working memory during a session, including an unresolved comparison encountered an hour earlier, should register in it quickly. Vigor sits between the poles, since energy and resilience accumulate over days and respond to both physical recovery and belief. Dedication is the slowest to form and the slowest to erode, resting on an identity investment that adolescents in organized training typically build over seasons rather than sessions. In youth athletic populations, these differences are pronounced. A 15-year-old may arrive at practice physically depleted yet remain proud of being an athlete, or train with obvious energy while mentally replaying a rival's posted result. Because the facets co-vary but are not interchangeable, treating them separately keeps the framework honest about where any upstream influence actually lands (Fredricks et al., 2004), and it generates the expectation, tested formally in Section 4.3, that fluctuation should reach absorption first and dedication last.

We argue that the link from online social feedback to engagement is not direct but routed through the efficacy state developed in Section 2.1. A like, a comparison, or an evaluative comment does not, by itself, add or subtract training energy; it adjusts the athlete's momentary belief about what they can accomplish, and that revised belief is what shows up as altered vigor or sharpened focus (Anderman and Patrick, 2012). This mediational logic follows from social cognitive theory's claim that environmental inputs reach behavior through cognitive appraisal rather than around it (Bandura, 2004). Feedback that nudges δ_*i,t*_ upward should propagate into stronger engagement; feedback that pushes it down should drain the same facets.

Yet the same feedback does not move every adolescent equally, and the most defensible reason is dispositional rather than situational. Social comparison orientation, the stable tendency to attend to and define oneself against others' standing, governs how forcefully a given comparison event is registered (Gibbons and Buunk, 1999). A high-orientation athlete scrutinizes peer performance closely, so an upward comparison bites harder and the efficacy excursion is larger; a low-orientation athlete lets the same signal pass with little internal consequence (Buunk and Gibbons, 2007). This makes comparison orientation a moderator of the feedback-to-efficacy link rather than a separate cause, and positioning it there lets the model explain why identical online environments yield divergent psychological trajectories.

Combining these pieces, the conditional indirect effect of an online feedback input *X* on engagement *Y* through efficacy fluctuation *M*, moderated by comparison orientation *W*, can be expressed in the conventional moderated-mediation form shown in Equation 2 (Hayes, 2013):


$$\begin{array}{l} Y=\beta_{0}+\left(\;a_{1}+a_{3}W\;\right)b\;X+c^{\prime }X+\varepsilon \end{array}$$ (2)

where *a*_1_ is the baseline feedback-to-efficacy path, *a*_3_ the interaction with comparison orientation, *b* the efficacy-to-engagement path, and *c*′ the residual direct effect. The product (*a*_1_ + *a*_3_*W*)*b* is the quantity the analysis later estimates, and its dependence on *W* is precisely the moderated mediation we claim. Because the same symbols recur throughout, every coefficient is defined at first appearance, and the notation is not redefined for later equations.

Figure 2 presents the full structure and is drawn in two tiers so that the level of each path is legible on the page. The lower tier carries the within-person process, in which occasion-level feedback *Feedback*_*i,t*_ moves the momentary deviation δ_*i,t*_. The upper tier carries the between-person structure, in which comparison orientation *W*_*i*_, trait efficacy and the person-level amplitude indices stand as athlete-to-athlete differences that predict weekly engagement. The moderator crosses the two tiers, since a person-level disposition conditions an occasion-level slope. Keeping the engagement facets disaggregated in the diagram is deliberate, since one empirical question worth asking is whether efficacy fluctuation reaches vigor, dedication, and absorption through the same coefficient or three distinct ones (Fredricks and McColskey, 2012).

**Figure 2:**
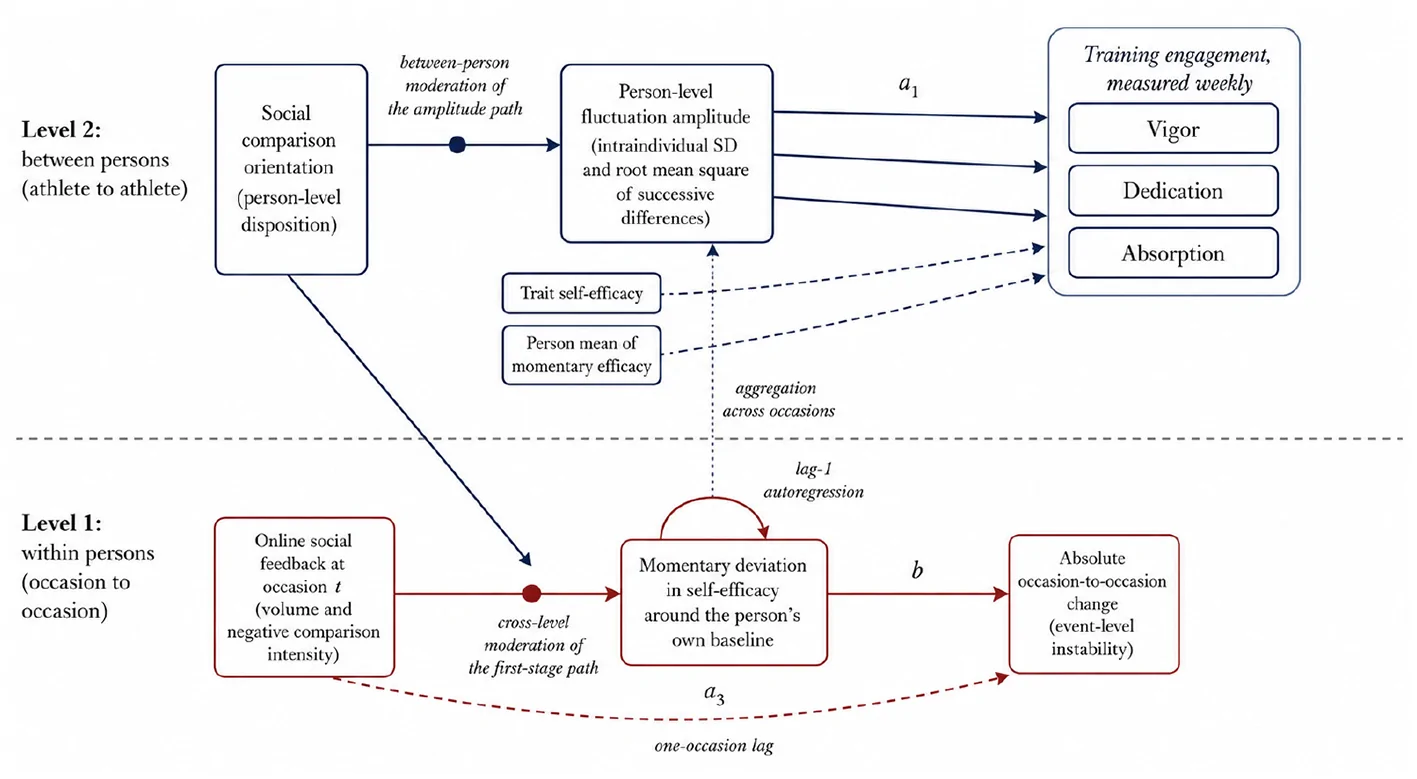
Two-tier conceptual model separating the within-person process, in which occasion-level online social feedback drives momentary deviations in self-efficacy, from the between-person structure, in which person-level fluctuation amplitude, trait efficacy, and comparison orientation relate to the three facets of training engagement, with comparison orientation shown as a cross-level moderator of the first-stage path.

Building on this reasoning, we advance three level-based hypotheses. H1: online social feedback predicts adolescent athletes' training engagement, with negatively valenced comparison feedback depressing vigor, dedication, and absorption, each tested separately rather than on a composite. H2: self-efficacy fluctuation mediates this relationship, carrying the feedback effect into the engagement facets. H3: social comparison orientation moderates the first stage of the mean efficacy path, such that the feedback-to-efficacy association strengthens as orientation rises, producing a conditional indirect effect on engagement.

### 2.3 Statement of research hypotheses

Before stating the remaining propositions, it is worth making explicit why the inquiry does not stop at H1 through H3. Mean-level hypotheses answer a question about position: where, on average, does an athlete's confidence sit, and does the average shift with the tone of the feed. They say nothing about a question of form: how that confidence moves between one measurement and the next. Two athletes can share a mean and differ entirely in their trajectories: one holding a steady line and the other oscillating through wide arcs, and the distinction is not cosmetic, because the temporal signature carries information about regulatory capacity (Kuppens and Verduyn, 2017). Averaging is, in this sense, not a neutral summary but a deletion. The volatility hypotheses that follow are therefore additive rather than substitutive: they do not claim that the mean is uninformative, and each of them is tested with mean efficacy retained as a covariate so that any incremental contribution is visible. The two frameworks may also interact, in that a wide swing should cost more when it descends from an already low baseline than from a high one, and the control proposition in Table 1 that mean efficacy positively predicts engagement is what allows that comparison to be made.

**Table 1:** Summary of research hypotheses with corresponding construct relationships and theoretical grounding.

Hypothesis	Proposed relationship	Theoretical basis
H1	Negative comparison feedback depresses vigor, dedication, and absorption, tested facet by facet	Social cognitive theory; engagement structure
H2	Self-efficacy fluctuation mediates the feedback–engagement relationship	Cognitive appraisal mediation in SCT
H3	Comparison orientation moderates the first-stage feedback-to-mean-efficacy path	Social comparison orientation theory
H4	Momentary self-efficacy shows a non-zero within-person lag-1 autoregressive parameter	State–trait distinction; intensive longitudinal modeling
H5a	Greater feedback volume predicts larger fluctuation amplitude, mean efficacy controlled	Source-density account of efficacy revision
H5b	Greater negative comparison intensity predicts larger amplitude, independently of volume	Negativity weighting in adolescent social cognition
H6	Fluctuation amplitude negatively predicts engagement beyond mean efficacy	Belief-instability and self-regulation theory
H7	Comparison orientation amplifies the negative-intensity-to-amplitude association	Moderated dispositional reactivity
–	Mean self-efficacy positively predicts engagement (baseline control)	Classical self-efficacy theory
–	Engagement facets respond heterogeneously to efficacy change	Multidimensional engagement framework

The first temporal proposition concerns the form of self-efficacy itself. If the state component δ_*i,t*_ behaves as Section 2.1 supposes, then an adolescent athlete's confidence should not read as a flat line punctuated by random scatter but as a series with genuine temporal structure, where today's deviation carries information about tomorrow's (Hamaker and Wichers, 2017). Detecting such autocorrelation matters because it separates meaningful dynamics from measurement noise, and it licenses the time-series modeling taken up later. This yields H4: momentary self-efficacy exhibits non-random fluctuation with a significant within-person lag-1 autoregressive parameter rather than independent occasion-to-occasion variation.

A second cluster ties the online environment to the amplitude of that fluctuation, and the claim must be stated more finely than a single word allows if it is to be tested cleanly. The word exposure conflates two distinct quantities: how much evaluative feedback an athlete receives, and how unfavorable that feedback is. Because the analytic model separates volume from valence, the hypothesis should do the same. We therefore state H5 in two parts, both between-person claims about the amplitude indices and both estimated with mean efficacy controlled. H5a holds that greater feedback volume, the sheer density of posting, reactions, and comparison-relevant browsing, predicts larger fluctuation amplitude, on the reasoning that denser evaluative input supplies more frequent occasions for the belief to be revised (Kuppens et al., 2010). H5b holds that greater negative comparison intensity predicts larger amplitude independently of volume (Houben et al., 2015). Separating the two matters because they can dissociate: an athlete may receive a great deal of feedback that is overwhelmingly affirming.

The third concern returns to consequences. Fluctuation, not merely mean efficacy, is claimed to affect engagement, and this needs its own statement because the two can diverge, since an athlete with healthy average confidence but violent swings may still train erratically (Vallerand, 1997). H6 holds that the amplitude of self-efficacy fluctuation is negatively associated with training engagement across all three facets, beyond the effect of mean efficacy. Pairing H6 with the mediational claim of H2 lets us ask whether instability is a distinct route or merely a restatement of low average belief (Trull et al., 2015).

Finally, the moderating role of comparison orientation deserves restatement at the level of fluctuation rather than mean, and it must be kept separate from H3. Athletes high in comparison orientation should not only react more strongly on average but should also display larger amplitude when placed in comparison-rich feeds (Buunk et al., 1990). H7 therefore proposes that social comparison orientation amplifies the between-person association between negative comparison intensity and fluctuation amplitude. H3 and H7 locate moderation on different paths, the first on the mean efficacy path of Equation 2 and the second on the amplitude path, and Section 4.3 accordingly reports an index of moderated mediation for each rather than one index for both.

Table 1 collects these propositions alongside their theoretical anchoring. As presented in Table 1, each hypothesis maps to a specific construct relationship and the conceptual source from which it is derived (Baron and Kenny, 1986).

Because level of analysis determines both what an estimate means and what may legitimately be inferred from it, Table 2 states for each hypothesis the level at which it is framed, the equation and model that test it, and the table reporting the result. As Table 2 shows, H4 and the lagged effects are within-person claims, H5 through H7 are between-person claims about person-level amplitude, and H1 through H3 involve paths that cross the two levels; reading the results against this map is the simplest guard against treating a between-person association as though it described a process unfolding inside an individual (Preacher et al., 2010).

**Table 2:** Alignment of each hypothesis with its level of analysis, estimating model, and reporting location.

Hypothesis	Level of the claim	Model and section	Reported in
H1	Occasion-level antecedent, person-level outcome (1 → 2)	Multilevel SEM, Section 3.3	Table 9
H2	Cross-level indirect path (1 → 2 → 2)	Multilevel SEM, Section 3.3	Table 9
H3	Person-level moderator on an occasion-level slope	Cross-level interaction, Section 3.3	Table 7
H4	Within-person (Level 1)	Two-level DSEM, Section 3.3	Table 6
H5a, H5b	Between-person (Level 2)	Amplitude regression, Section 3.3	Table 8
H6	Between-person (Level 2)	Multilevel SEM, Section 3.3	Table 9
H7	Between-person moderation of a Level-2 slope	Amplitude regression, Section 3.3	Table 8
Lagged multilevel model, Section 4.2	Within-person (Level 1)	Lagged multilevel model, Section 4.2	Table 7

Taken together, the seven hypotheses move the inquiry from level to dynamics. H1 through H3 retain the conventional level-based logic; H4 through H7 reframe the same constructs in terms of temporal variability, which is where the more revealing evidence is expected to lie. The two control propositions in the lower rows of Table 1 are not formal hypotheses but anchoring expectations that, if violated, would signal a misspecified model rather than a substantive finding.

## 3 Methods and data sources

### 3.1 Study design and momentary data collection

The questions posed in Section 2 cannot be answered by a one-shot survey, so we adopted a mixed design pairing ecological momentary assessment with a longitudinal follow-up backbone (Shiffman et al., 2008). The momentary layer captures self-efficacy and recent online social activity at a fine temporal grain, whereas the follow-up layer anchors those readings to slower-moving traits and to training engagement assessed at fixed intervals. The logic of the pairing is straightforward: fluctuation demands dense within-day sampling, whereas the mediational and engagement claims need stable endpoints measured across weeks (Stone and Shiffman, 1994). Over a continuous eight-week window, participants responded to four signal-contingent prompts per day delivered through a smartphone questionnaire, supplemented by weekly engagement assessments and a baseline trait battery.

Prompt scheduling followed the recommendations for athlete samples set out by Reifsteck and colleagues (Reifsteck et al., 2021). The day was divided into four fixed windows, 09:00–11:00, 13:00–15:00, 17:00–19:00 and 20:30–22:00, and within each window the signal was delivered at a random minute, so that responses were neither predictable nor systematically tied to a single point in the training day. Participants had 30 mins to respond before a prompt expired. Because most squads trained in the late afternoon, the third window typically fell shortly after a session and the fourth in the evening period when platform use peaks, while the first two fell before training. This arrangement deliberately spreads observations across pre-training, post-training, and high-exposure evening periods rather than concentrating them in one; it does mean that post-training and post-competition states are represented in roughly a quarter of occasions, a point we return to when interpreting the calendar-linked pattern in Section 4.1. We did not exclude competition days since they are part of the ecology under study. Two properties of this schedule bound what the data can settle, and we prefer to state them here and address them analytically later rather than file them as caveats. Fixed windows with a randomly timed signal inside them capture the day as it is ordinarily lived, but they cannot guarantee that the most charged moments—the 20 mins after a result is announced, say—fall inside a window; the design is signal-contingent, not event-contingent. We did, however, record for every athlete and every week the phase of the competitive calendar assigned by the coaching staff, distinguishing ordinary training weeks, the high-load block preceding regional fixtures, competition weeks, and post-competition recovery weeks. That schedule is the most obvious candidate for a common cause, because the weeks that concentrate unfavorable comparison also concentrate training load, fatigue, and result-related stress. We did not collect objective indicators of that load: session ratings of perceived exertion, wearable-derived external load, competition placings, or recovery scores (Impellizzeri et al., 2019). Calendar phase is a coarse stand-in for all of these, and Section 3.3 explains how we use it.

Recruitment proceeded through youth sport training programs, with athletes sampled by a stratified approach across discipline, competitive tier, and sex to avoid a sample skewed toward any single sport culture (Tenenbaum and Eklund, 2007). Eligibility required active enrollment in organized training, regular use of at least one social platform, and an age between 13 and 18. The distinction between individual and team disciplines was retained in the stratification and is reported in Table 3, because the density of comparison referents and the character of feedback plausibly differ between a swimmer whose result is a public number and a midfielder whose contribution is diffuse. Competitive tier was treated the same way, and for a parallel reason. A national program and a school squad may differ not only in how much comparison-relevant material an athlete encounters but in what training engagement means to the athlete in the first place, so tier was not left as a description of the sample. It does not enter the baseline models, whose specification was fixed in advance, but we re-estimated every multilevel and structural model with tier as a Level-2 covariate and its interaction with negative comparison intensity; we report those results once, in Section 4.3. The national tier remains the smallest of the three groups, which limits the power of that test and is revisited among the limitations.

**Table 3:** Demographic characteristics of the analytic sample.

Characteristic	Category	*n*	%	Mean (SD)
Sex	Male	168	52.5	–
Sex	Female	152	47.5	–
Age (years)	13–15	147	45.9	15.2 (1.4)
Age (years)	16–18	173	54.1	–
Competitive tier	Recreational/school	121	37.8	–
Competitive tier	Regional/provincial	142	44.4	–
Competitive tier	National	57	17.8	–
Sport type	Individual	179	55.9	–
Sport type	Team	141	44.1	–
Training history (years)	–	320	100	4.6 (2.3)

The research team collected all momentary, weekly, and baseline records directly through a self-hosted instrument; no proprietary third-party datasets were purchased or scraped. What is open in this study, to restate the point made in the Introduction, is the instrumentation and software rather than the data source. Figure 3 lays out the full sequence from recruitment through anonymization, so that each stage of the workflow can be audited and, in principle, repeated.

**Figure 3:**
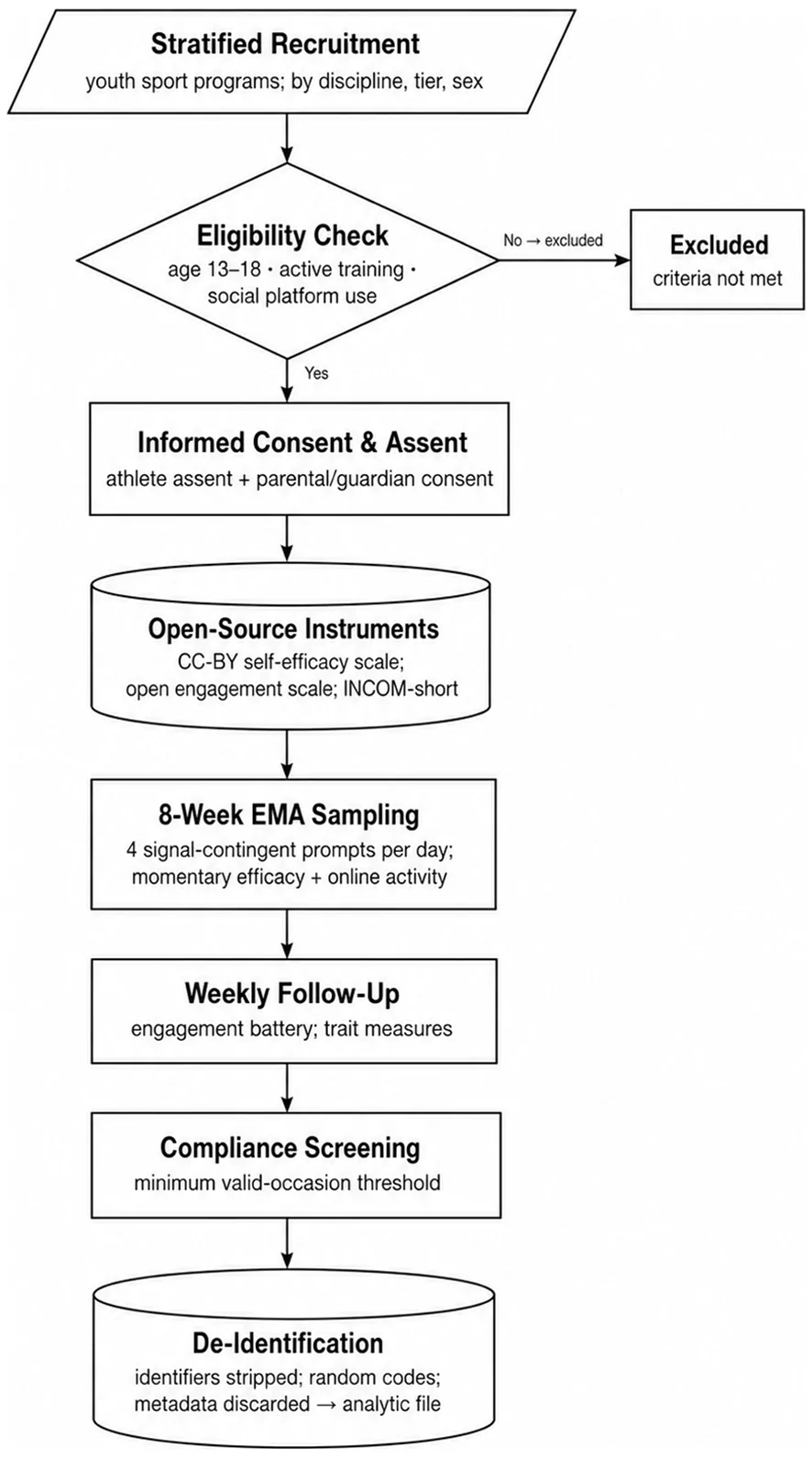
Workflow of the mixed ecological momentary assessment and longitudinal follow-up design, tracing the path from stratified recruitment and informed consent through the 8-week signal-contingent sampling, weekly engagement measurement, and the de-identification step applied before analysis.

We screened compliance before computing any index. Of the 224 scheduled prompts per athlete, we retained responses only for participants completing at least 112, that is, half of the scheduled total, a threshold chosen because dispersion estimates from sparser series are unstable enough to distort rather than inform (Jones et al., 2019). Six recruited athletes fell below the threshold, and a further 26 withdrew or were lost to follow-up, leaving the 320 athletes described in Table 3. Among the retained sample, the overall response rate was 79.1%, giving a mean of 3.16 responses per day (SD = 0.46) and a mean of 6.36 days with at least one valid response per week (SD = 0.25). The median athlete supplied 184 valid occasions, with a range from 114 to 216. Figure 4 shows both the distribution of completed prompts and its stability across the 8 weeks; compliance did not decline appreciably over the study window, which matters because a systematic drift would confound the trajectory results reported later.

**Figure 4:**
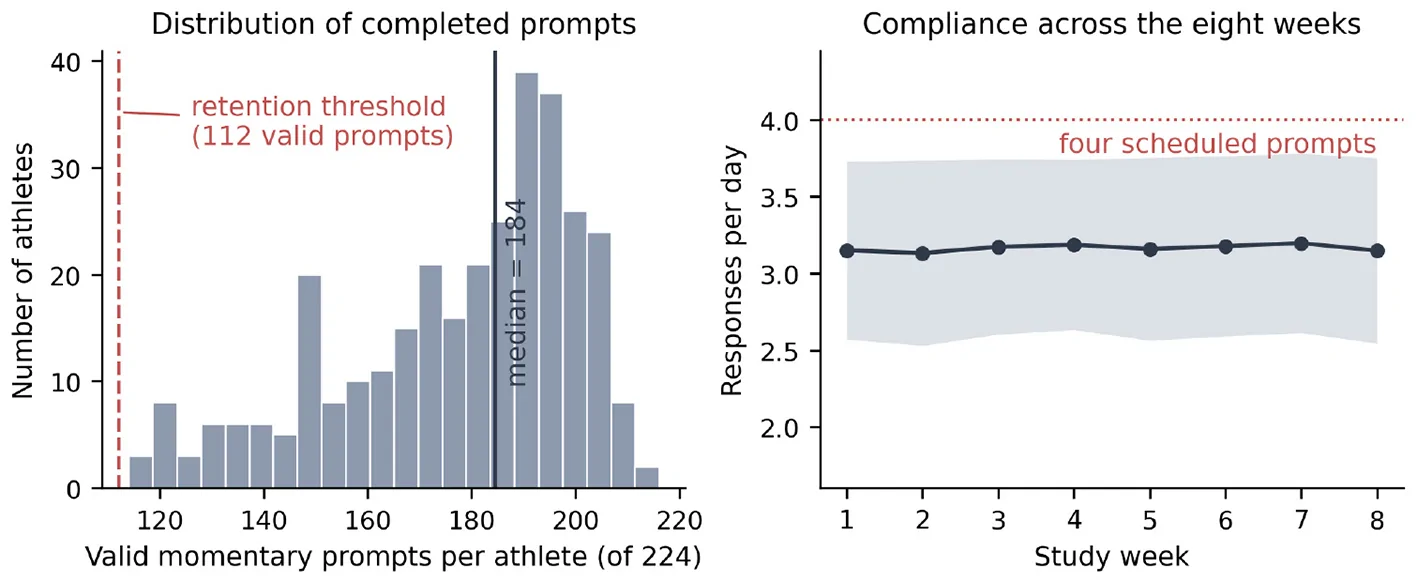
Compliance profile of the momentary sampling protocol, showing the distribution of valid prompts completed by each retained athlete against the retention threshold and the median, and the mean number of daily responses across the 8-week window with one standard deviation shaded.

We treated missing occasions as missing at random conditional on the modeled covariates and handled them using full-information maximum likelihood in the multilevel and structural models, without imputing momentary scores. Because prompts were delivered at random minutes within fixed windows, the intervals between consecutive responses are irregular; the dynamic models described in Section 3.3 therefore treat time explicitly rather than assuming equal spacing, and successive differences spanning a break of more than one calendar day were excluded from the sequential index.

The sample retained for analysis is described in Table 3. As shown in Table 3, the final set spans both sexes, the full adolescent age band, a range of competitive tiers and training histories, and both individual and team disciplines, supporting cautious generalization across the youth-athlete population rather than a single niche.

Ethical handling shaped the design throughout rather than being appended to it. The protocol received institutional review board approval as a study of healthy adolescents using questionnaire-based educational and behavioral measures, with no clinical intervention (World Medical Association, 2013). Because participants were minors, we obtained informed assent from the athletes and written consent from a parent or guardian before sending any prompts. Direct identifiers were stripped at intake and replaced with random codes, device-level metadata were discarded, and only de-identified momentary records entered the analytic files, steps that protect a population already exposed to the visibility pressures this study set out to examine.

### 3.2 Variable measurement and construction of fluctuation indices

Each construct carries its own measurement burden, and we checked reliability at the level where the variable operates rather than assuming it from a published coefficient. Trait self-efficacy, trait engagement, and social comparison orientation were scored from the baseline battery and evaluated with Cronbach's α and McDonald's ω, the latter preferred when item loadings were uneven (McDonald, 1999). The four-item momentary form demands a within-person reliability logic instead, so we estimated multilevel reliability from a two-level confirmatory factor model with occasions nested in persons, which partitions item covariance into within- and between-person components and yields a coefficient describing how consistently the short form behaves moment to moment rather than how well it separates individuals (Geldhof et al., 2014). Table 4 reports the instruments, their facet structure, and the coefficients obtained in this sample.

**Table 4:** Measurement of study variables and reliability coefficients.

Variable	Measurement source	Items/structure	Reliability (α/ω)
Trait self-efficacy	Baseline CC-BY efficacy scale	10 items, unidimensional	0.89/0.90
Momentary self-efficacy	EMA brief efficacy form	4 items, signal-contingent	0.82 (within-person ω)
Training engagement	Open engagement scale	17 items, 3 facets	0.91/0.92
- Vigor	Engagement subscale	6 items	0.85/0.86
- Dedication	Engagement subscale	5 items	0.88/0.88
- Absorption	Engagement subscale	6 items	0.83/0.84
Comparison orientation	Public-domain INCOM-short	6 items	0.80/0.81

As shown in Table 4, all multi-item scales cleared conventional thresholds, and the momentary form held together adequately at the within-person level, which gave us enough confidence to build dispersion measures on top of it. The instruments themselves were deliberately chosen from those released under permissive open licenses, both to respect provenance and to let others reuse the exact item set: momentary and trait self-efficacy were measured with an openly available efficacy scale distributed under a CC-BY license and adapted to a brief momentary form (Schwarzer and Jerusalem, 1995), training engagement with a freely redistributable engagement scale covering the three facets, and comparison orientation with a short public-domain measure. All efficacy and engagement items used a seven-point response format anchored at 1 and 7, so every index reported below is expressed in those scale points unless explicitly standardized; the axes of the figures are labeled accordingly.

Online social feedback could not be read off a single item, so we coded it from the momentary reports of platform activity gathered at each prompt. For each occasion at time *t*, participants indicated counts of posting, received reactions, comments, and comparison-relevant browsing over the preceding interval. Two distinct quantities were then formed. Feedback volume is the standardized sum of these counts, indexing sheer density irrespective of tone. Negative comparison intensity is the standardized count of events the participant flagged as unfavorable, that is, occasions on which they reported encountering a peer performing better, receiving criticism, or seeing a reaction they judged disappointing relative to expectation. Valence was therefore participant-coded at the moment of report rather than inferred afterward, which avoids the interpretive problems of *post-hoc* rating but makes the coding rule worth stating precisely: each reported event was classified into a positive, negative, or neutral category by a single forced-choice item, and events left neutral entered volume but not intensity. To check that the rule was applied consistently, two trained assistants independently recoded the free-text descriptions accompanying a random 15% of flagged events; agreement with the participant classification was high (Cohen's κ = 0.84), and the residual disagreements concerned ambiguous humor rather than the valence categories themselves. Keeping volume distinct from intensity matters because H5a concerns density while H5b and H1 concern direction, and conflating them would blur the two. Two limits of the rule deserve to be named where the rule itself is defined. A single forced-choice item carries the entire valence judgment. That judgment is made by the very person whose disposition we later treat as a moderator, so an athlete high in comparison orientation may simply be readier to file an ambiguous remark under the negative heading. Rather than concede the point and move on, we put a check on it: the free-text descriptions were scored with a dictionary-based sentiment procedure of the kind developed for psychological text analysis (Tausczik and Pennebaker, 2010), and we asked whether the agreement between automated polarity and participant valence itself shifted with comparison orientation. Section 4.3 reports the answer.

The fluctuation indices are the analytic core of this study, and we constructed two complementary dispersion measures from the repeated momentary efficacy scores. The first is the intraindividual standard deviation, capturing the overall spread of an athlete's readings around their own mean. For person *i* with *T*_*i*_ valid occasions, the intraindividual standard deviation is calculated using Equation 3:


$$\begin{array}{l} iSD_{i}=\sqrt{\frac{1}{T_{i}-1}\overset{T_{i}}{\underset{t=1}{\sum }}{\left(SE_{i,t}-SE_{i}\right)}^{2}} \end{array}$$ (3)

The intraindividual standard deviation treats all deviations alike regardless of ordering, which is exactly its limitation: it sees scatter but not sequence. To recover the temporal texture, we added the root mean square of successive differences, a standard index of moment-to-moment instability that weights how sharply consecutive readings change, as shown in Equation 4 (Jahng et al., 2008):


$$\begin{array}{l} RMSSD_{i}=\sqrt{\frac{1}{T_{i}-1}\overset{T_{i}-1}{\underset{t=1}{\sum }}{\left(SE_{i,t+1}-SE_{i,t}\right)}^{2}} \end{array}$$ (4)

The two indices answer different questions and are retained for that reason, not as a robustness gesture. A high intraindividual standard deviation with a low root mean square of successive differences describes an athlete who drifts slowly between confidence levels; a high root mean square of successive differences describes one who oscillates abruptly between adjacent occasions. Conceptually, the sequential index more closely captures the dynamic essence of fluctuation and better matches the temporal structure of momentary data, while the dispersion index is more readily comparable across individuals and across studies using different sampling densities. On these grounds, the root mean square of successive differences is the single operationalization of amplitude entering the structural model of Section 4.3, and we treat it as primary throughout, because it is the index that speaks to a process unfolding in time and the mediation claim is a claim about such a process. The intraindividual standard deviation runs alongside it wherever a between-person regression is reported, and we re-estimated the structural model with it as a robustness check. Hence, no conclusion turns on the choice. Figure 5 contrasts the two profiles to make the distinction concrete.

**Figure 5:**
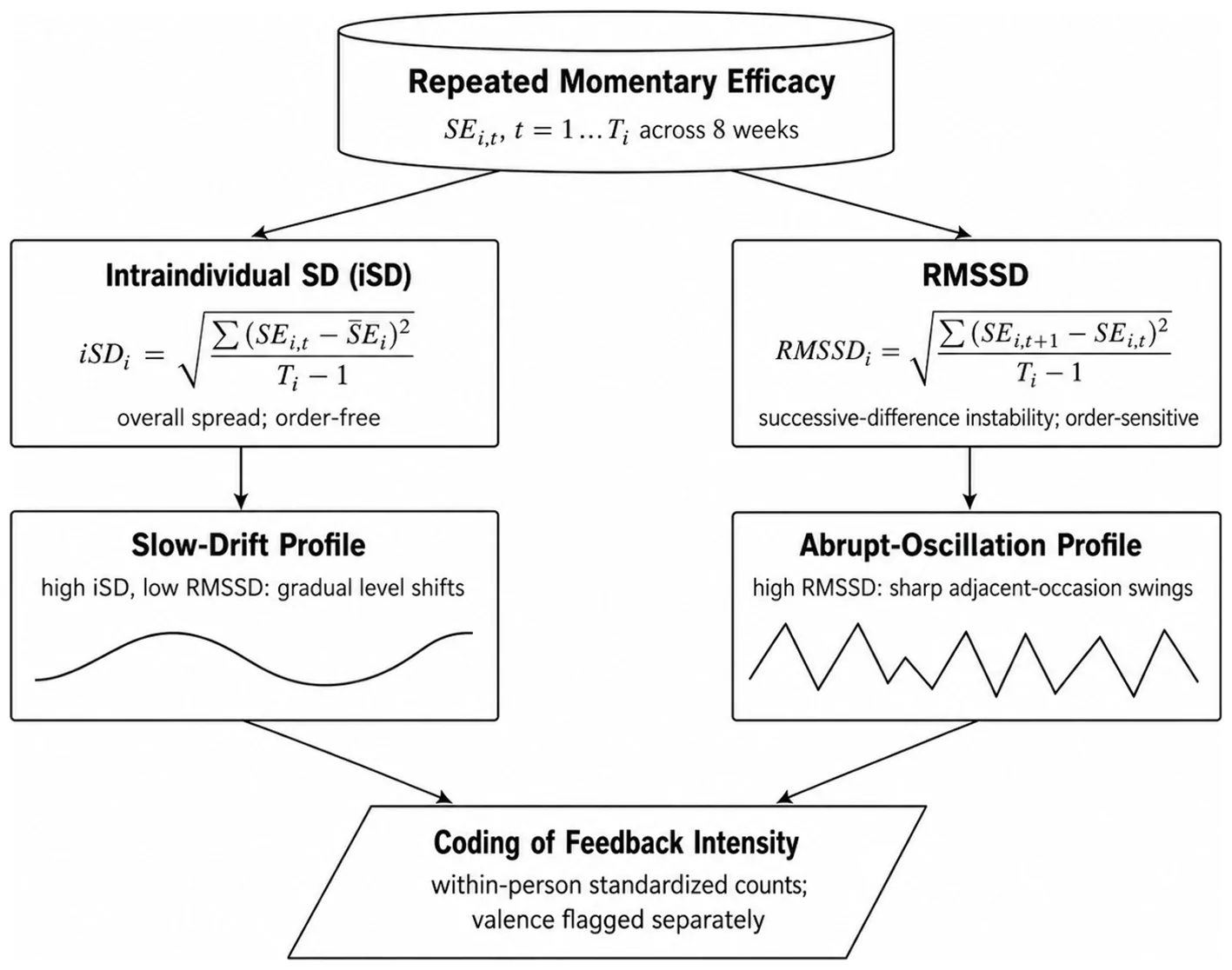
Schematic illustration distinguishing the two fluctuation indices, showing how intraindividual standard deviation captures overall dispersion while the root mean square of successive differences reflects the sharpness of occasion-to-occasion change in momentary self-efficacy.

One property of both indices requires explicit checking rather than assertion. On a bounded response scale, an athlete whose mean sits near the ceiling has less room to vary, so dispersion measures are arithmetically entangled with the mean, and any claim that amplitude matters beyond average confidence can be unstable (Dejonckheere et al., 2019). Figure 6 therefore plots each index against the person mean. In this sample, entanglement is mild: the mean correlates with the dispersion index at *r* = −0.18 and with the sequential index at *r* = −0.10, while the two indices correlate at *r* = 0.88 with one another. The negative sign is in the expected direction, and the magnitudes are small enough that the incremental tests reported in Section 4.3 are not being carried by collinearity, but the mean is nevertheless retained as a covariate in every amplitude model. The high correlation between the indices also explains, in advance, why substituting one for the other changes little. Controlling the mean is the conventional remedy, yet it is not the strictest one available. A dispersion index can be inflated from one side by a low mean and from the other by a sluggish, heavily autocorrelated series, so we additionally constructed a residualized amplitude score: the sequential index regressed on the person mean and on the person-specific lag-1 autoregressive parameter φ_*i*_, with the residual retained. The reasoning follows the case made for mean-corrected variability measures on bounded scales (Mestdagh et al., 2018). If the index were only scatter around a level, its residual should carry no further information about engagement; Section 4.3 reports what happens when the residual takes its place.

**Figure 6:**
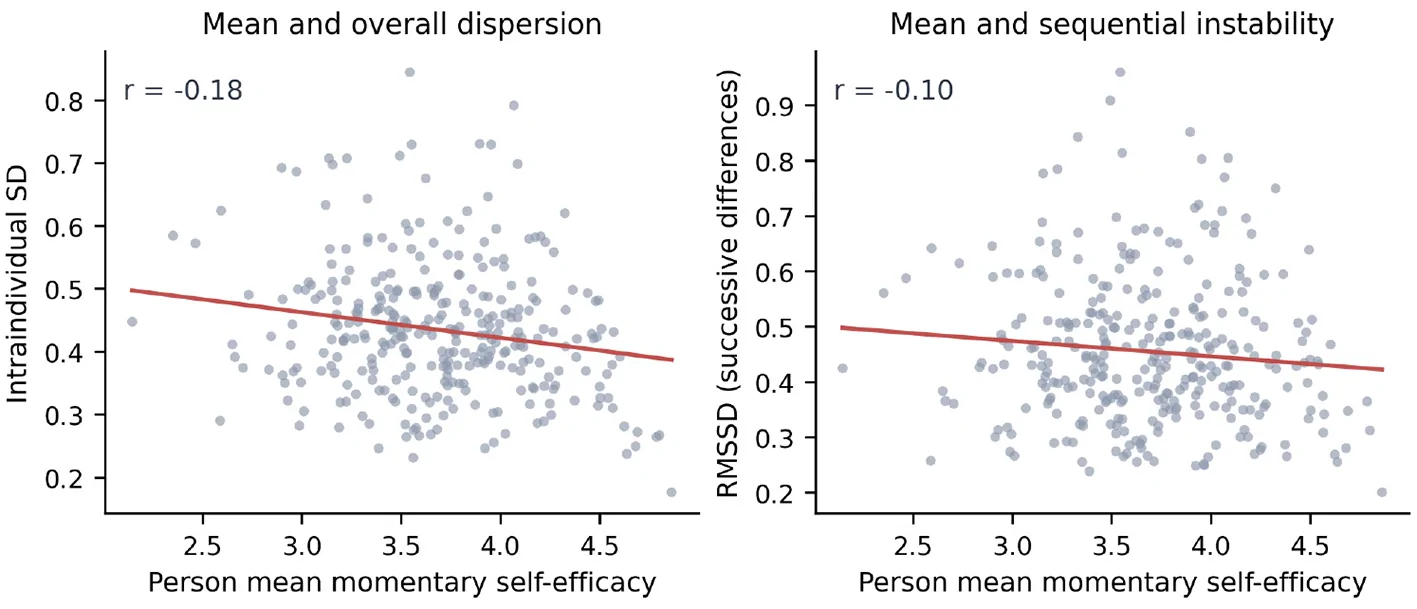
Association between the person mean of momentary self-efficacy and each fluctuation index, with the fitted linear trend and the corresponding correlation shown for the intraindividual standard deviation and for the root mean square of successive differences.

A final distinction of level must be flagged here because it governs the reading of Section 4.2. The two indices just defined are person-level summaries, trait-like in the sense that each athlete receives one value describing the character of their whole series. The absolute change between two adjacent readings, by contrast, is an event-level quantity: it describes one episode, varies within a person from occasion to occasion, and can be predicted by things that also vary within a person. Both indicate fluctuation, but they operate at different levels and answer different questions, and the terminology is kept fixed throughout the study accordingly. Fluctuation amplitude always refers to the person-level indices; momentary instability always refers to the event-level change; and the word fluctuation without qualification is reserved for the construct rather than any of its operationalizations.

### 3.3 Analytic strategy and open-source software pipeline

The nested shape of the data, occasions within days within persons, rules out any single-level regression, so the workhorse of the analysis is the multilevel linear model, which lets within-person processes and between-person differences be estimated at once without conflating them (Raudenbush and Bryk, 2002). For a momentary efficacy outcome predicted by time-varying feedback, the two-level specification takes the form shown in Equation 5:


$$\begin{array}{l} SE_{i,t}=\gamma_{00}+\gamma_{10}\;Feedback_{i,t}+\gamma_{01}\;W_{i}+\gamma_{11}\;Feedback_{i,t}\;W_{i}+u_{0i}\\ \;+u_{1i}\;Feedback_{i,t}+r_{i,t} \end{array}$$ (5)

where γ_10_ is the average within-person slope of efficacy on feedback, γ_11_ the cross-level interaction with comparison orientation *W*_*i*_ that tests H3, and *u*_0*i*_ and *u*_1*i*_ the random intercept and slope allowing each athlete their own baseline and reactivity (Singer and Willett, 2003). The occasion-level predictor is person-mean centered and the person mean is entered separately at Level 2, so the within- and between-person components of the association are not conflated. Random slopes were not a cosmetic addition: whether reactivity varies across athletes is itself an empirical question, so constraining it would have begged the point. Estimating interactions of this kind with non-normal observed data is not assumption-free, and the specification follows published recommendations for that case (Cham et al., 2012).

Testing H4 requires something the equation above does not provide, namely an estimate of temporal dependence within a person. We therefore fitted a two-level dynamic structural equation model in which the momentary score is decomposed into a person-specific mean and an autoregressive process around it (Asparouhov et al., 2018; Hamaker et al., 2018). The within-person part can be written as Equation 6:


$$\begin{array}{l} SE_{i,t}=\mu_{i}+\varphi_{i}\left(SE_{i,t-1}-\mu_{i}\right)+\zeta_{i,t} \end{array}$$ (6)

in which μ_*i*_ is the latent person mean, φ_*i*_ the person-specific lag-1 autoregressive parameter whose average and variance are estimated at Level 2, and ζ_*i,t*_ the innovation. Estimating μ_*i*_ as a latent quantity rather than substituting the observed person average matters more than it may appear; lagged within-person effects obtained from raw observed-mean centering are subject to a downward finite-sample bias. Because one of this study's headline results concerns a lagged effect, we report the dynamic model as the primary estimate and the conventional multilevel estimate alongside it for comparison (McNeish and Hamaker, 2020). We used the same framework to add the reverse path, from momentary efficacy to subsequent comparison-relevant browsing, which the original design left untested and which any directional reading of the results requires (Hamaker et al., 2015).

Capturing the shape of change over the 8 weeks called for a different lens. We fitted latent growth curve models to the weekly engagement series and person-period summaries of efficacy, which describe each athlete's trajectory through a latent intercept and slope rather than treating repeated measures as interchangeable (Duncan et al., 2006). In these models, the predictors of the growth parameters are person-level quantities, that is, an athlete's average feedback exposure and their comparison orientation, not time-varying covariates; the distinction is noted in the table so that the coefficients are not misread as occasion-level effects.

The hypothesized paths were then assembled into a multilevel structural equation model so that mediation and moderation could be evaluated within one estimable system rather than through a chain of separate regressions that would accumulate error at each link (Kline, 2015). Specifying the level of each path is not a formality here. The antecedent, negative comparison, is measured at the occasion level; the mediator, fluctuation amplitude, is a person-level index; and the outcomes, the three engagement facets, are person-level quantities measured weekly. The mediation is therefore of the 1 → 2 → 2 form and is estimated with an explicit within-between decomposition, because single-level approaches applied to Level-2 outcomes conflate the within- and between-person components of the indirect effect (Preacher et al., 2010). The conditional indirect effect specified in Section 2.2 was tested by estimating the product of the first-stage and second-stage coefficients, with the index of moderated mediation defined in Equation 7:


$$\begin{array}{l} IMM=a_{3}\times b \end{array}$$ (7)

whose departure from zero signals that the indirect route depends on comparison orientation. Because H3 and H7 place moderation on different paths, this index is computed separately for each, and separately for the positive and negative arms of feedback, so that the valence asymmetry reported in Section 4.2 is preserved rather than averaged away. Confidence bounds for these products were obtained by Monte Carlo resampling rather than assuming a normal sampling distribution, since the product of two coefficients is rarely symmetric (Preacher and Selig, 2012; MacKinnon et al., 2007). Magnitude was reported alongside significance using the ratio of the indirect to the total effect and a standardized mediation effect size, since a reliably non-zero indirect path says nothing by itself about how much of the relationship it carries (Preacher and Kelley, 2011).

The between-person claims of H5 and H7 require their own model, since neither concerns an occasion-level process. With the amplitude index as the outcome, the person-level specification is given in Equation 8:


$$\begin{array}{l} Amp_{i}=\pi_{0}+\pi_{1}Vol_{i}+\pi_{2}Neg_{i}+\pi_{3}W_{i}+\pi_{4}\left(Neg_{i}\times \;W_{i}\right)\\ \;+\pi_{5}\mu_{i}+e_{i} \end{array}$$ (8)

where *Vol*_*i*_ is feedback volume, *Neg*_*i*_ negative comparison intensity, *W*_*i*_ comparison orientation and μ_*i*_ the person mean of momentary efficacy, included so that H5 and H7 are tested with baseline confidence held constant as the hypotheses state. We also estimated the volume-by-orientation product, and fitted both the dispersion and sequential indices as outcomes. Four supplementary specifications were then fitted in the service of the inferences drawn later, and each is reported once, in Section 4.3. The first adds calendar phase as a time-varying Level-1 covariate to the lagged model and afterward splits the series by phase, so that the lagged effect can be inspected separately in the high-load block, the competition weeks, and the recovery weeks. The second substitutes the residualized amplitude score for the raw sequential index throughout the structural model. The third enters competitive tier as a Level-2 covariate and tests its interaction with negative comparison intensity. The fourth sets automated sentiment polarity against participant-coded valence on the free-text subset and asks whether their agreement depends on comparison orientation. None of the four is a post hoc rescue of a result; each was specified to answer a rival reading that the design itself invites.

Every step ran inside the R statistical environment, chosen as much for principle as for capability, since the entire pipeline rests on freely licensed packages that any researcher can reinstall at no cost. Multilevel models were estimated with restricted maximum likelihood in the lme4 framework; growth curves and the structural model in lavaan; the dynamic model through an openly available Bayesian time-series implementation with 40,000 post–warm-up iterations across two chains and convergence judged by potential scale reduction factors below 1.05; and the moderated-mediation resampling through an openly available add-on. Package versions and the random seed are reported in full, and the de-identified data, the analysis scripts, and the complete unrounded model output are provided in the Supplementary material so that every estimate can be reproduced independently. Figure 7 summarizes how the four modeling layers map onto the hypotheses and onto the open-source software used at each stage.

**Figure 7:**
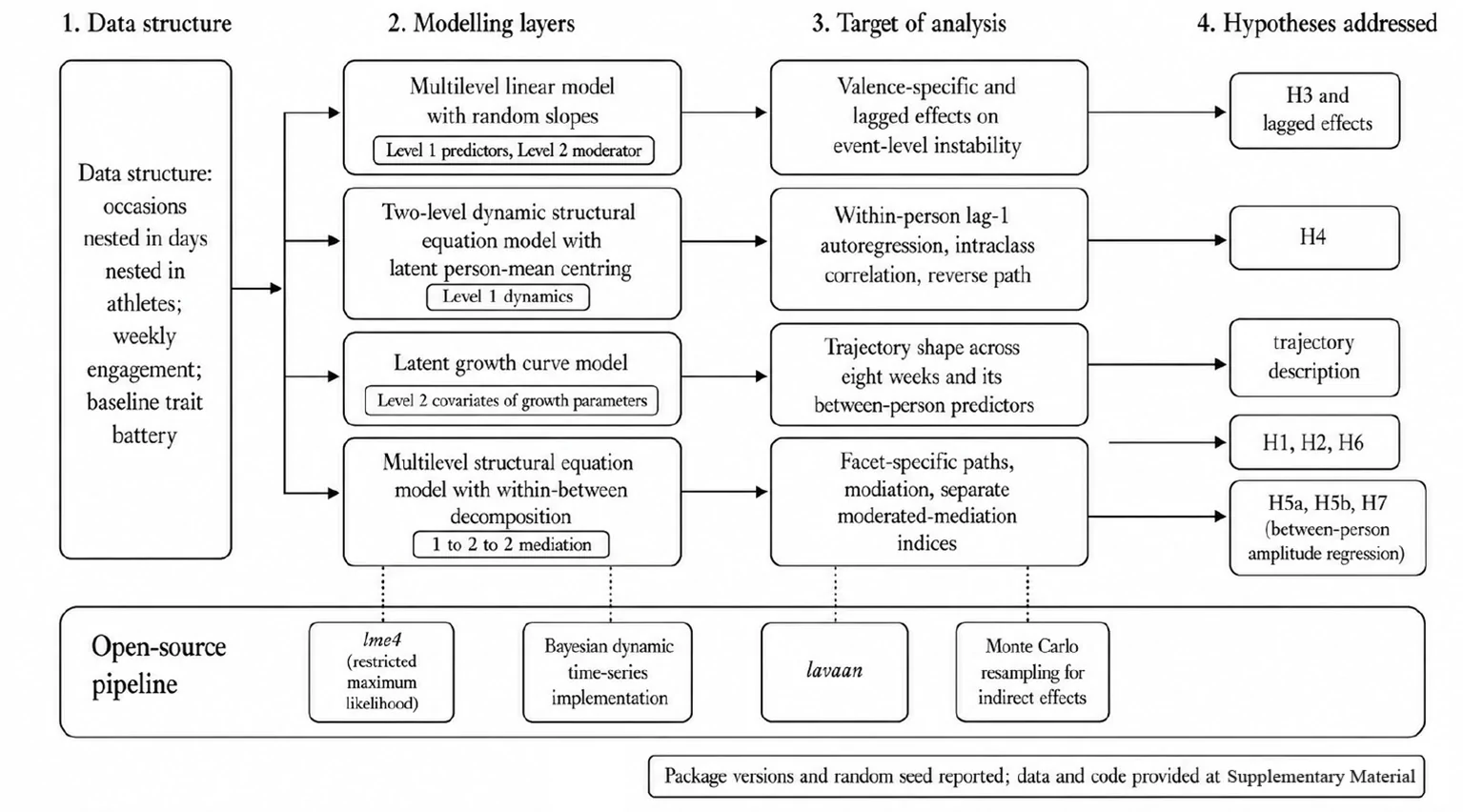
Architecture of the analytic pipeline, mapping the multilevel model, the two-level dynamic structural equation model, the latent growth curve model and the multilevel structural equation model onto the temporal, trajectory and path hypotheses respectively, with the open-source R packages used at each stage indicated alongside and each path labeled by its level of analysis.

## 4 Results

### 4.1 Characterizing the temporal regularities of self-efficacy fluctuation

The data make clear that adolescent self-efficacy over 8 weeks is anything but flat. Averaged across the sample, momentary efficacy traced a gently undulating course rather than a stable plateau, with a shallow mid-period dip and a partial recovery toward the close of the tracking window. Figure 8 plots the group-mean trajectory together with a band of individual series. The band is more informative than the mean line: athletes who shared almost the same average confidence followed visibly different paths, some nearly level and others swinging through wide arcs across adjacent weeks (Almeida, 2005).

**Figure 8:**
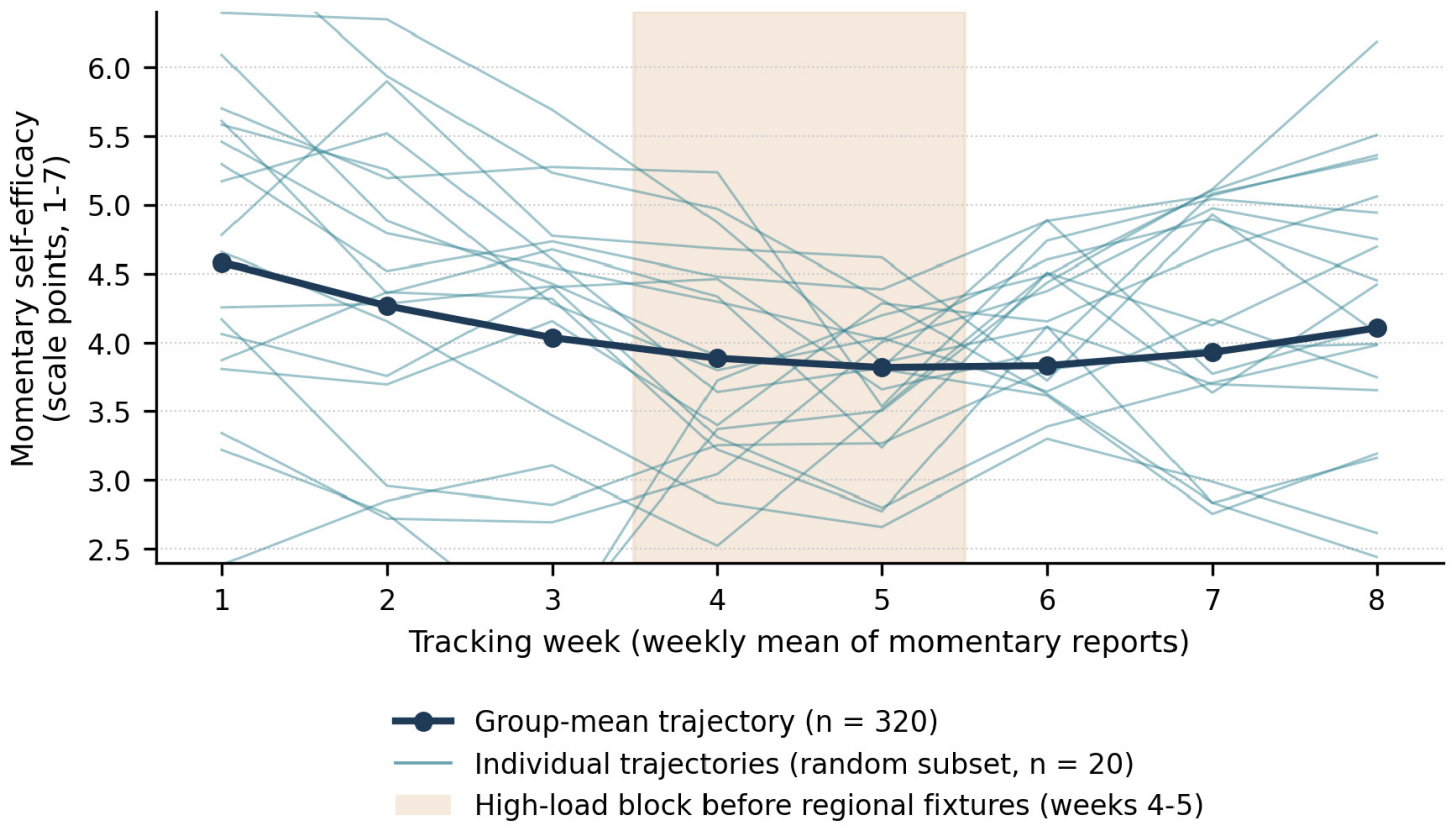
Group-mean weekly trajectory of momentary self-efficacy across the eight-week tracking period (*n* = 320), overlaid with a randomly drawn subset of twenty individual trajectories. Both axes are in the units of measurement: the vertical axis reports scale points on the seven-point momentary efficacy form, and the horizontal axis reports the tracking week, each point being the mean of that athlete's momentary reports within the week. The shaded band marks weeks 4 and 5, which the coaching calendar designated a high-load block preceding regional fixtures. The aggregate dip is modest; the heterogeneity of the individual series around it is not.

Fitting the latent growth curve confirmed that this heterogeneity is structural rather than incidental. The unconditional model returned a reliable latent intercept and a small negative average slope, and the variance components around both were sizeable, meaning individual athletes departed substantially from the average trajectory in both starting level and direction of change (Grimm et al., 2017). Table 5 reports the estimates. As presented in Table 5, the significant intercept and slope variances justify treating the trajectory parameters as quantities that themselves vary across persons. It is worth being precise about what this does. It is worth being precise about what this does: variance in growth parameters indexes heterogeneity in trajectories across athletes, a between-person property, and the intercept of 3.84 describes an average level rather than a dispersion. Neither quantity speaks to within-person temporal dependence, which H4 asserts and the model reported next actually tests.

**Table 5:** Latent growth curve model estimates for momentary self-efficacy across the tracking period.

Parameter	Estimate	SE	z	*p*
Intercept (mean)	3.842	0.061	62.98	<.001
Slope (mean)	−0.068	0.019	−3.58	<.001
Quadratic (mean)	0.041	0.011	3.73	<.001
Intercept variance	0.407	0.069	5.90	<.001
Slope variance	0.089	0.020	4.45	<.001
Intercept–slope covariance	−0.063	0.021	−3.00	.003
Residual (occasion)	0.334	0.031	10.77	<.001
Mean feedback exposure on slope (Level 2)	−0.118	0.039	−3.03	.002
Comparison orientation on slope (Level 2)	−0.092	0.031	−2.97	.003

Feedback exposure and comparison orientation enter as between-person (Level 2) covariates of the latent growth parameters, not as time-varying covariates.

The reliable quadratic term deserves comment, because it points to phasing rather than steady drift. The mid-period trough aligned with a stretch the training calendar marked as a high-load block preceding regional fixtures, and swings widened around those competitive nodes before settling once results were known (Kellmann, 2010). Periods of heavy online activity, clustered predictably just after competition when athletes posted and compared most, coincided with the sharpest occasion-to-occasion movement, an association we read as descriptive rather than causal at this stage. The negative coefficient for mean feedback exposure on the growth slope, shown in the lower rows of Table 5, indicates that athletes with denser online exposure tended to follow a more downward trajectory. However, this is a between-person comparison and disentangling it from the competitive cycle is work for the models that follow. Whether that coincidence, rather than comparison itself, does the explanatory work is precisely the question the phase-based sensitivity analyses of Section 4.3 were built to probe.

Turning to H4 itself, the two-level dynamic model estimated a clearly non-zero lag-1 autoregressive parameter: momentary efficacy at one occasion carried substantial information about the next, and the parameter varied appreciably across athletes. Table 6 reports the estimates. As Table 6 shows, the pooled autoregressive parameter of 0.44 with a credible interval well clear of zero settles the descriptive question that H4 posed, and the intraclass correlation of 0.53 indicates that roughly half the variance in momentary readings lies between athletes and half within them, which is what makes both levels of analysis worth pursuing. The comparison in the lower rows is instructive in its own right: the same dependence estimated occasion by occasion without latent person-mean centering comes out at 0.31, an attenuation of the expected direction and magnitude (McNeish and Hamaker, 2020). Figure 9 displays the distribution of individual estimates against both pooled values.

**Table 6:** Two-level dynamic structural equation model of within-person temporal dependence in momentary self-efficacy (Level 1), with the uncorrected occasion-level estimate shown for comparison.

Parameter	Estimate	SE/posterior SD	95% interval
Lag-1 autoregression, pooled (latent centering)	0.443	0.005	[0.432, 0.454]
Between-person SD of the autoregression	0.066	0.009	[0.049, 0.084]
Within-person innovation variance	0.281	0.011	[0.260, 0.303]
Between-person variance of the person mean	0.318	0.027	[0.267, 0.373]
Intraclass correlation of momentary efficacy	0.526	0.023	[0.481, 0.571]
Lag-1 autoregression, observed-mean centering	0.311	0.006	[0.299, 0.323]

**Figure 9:**
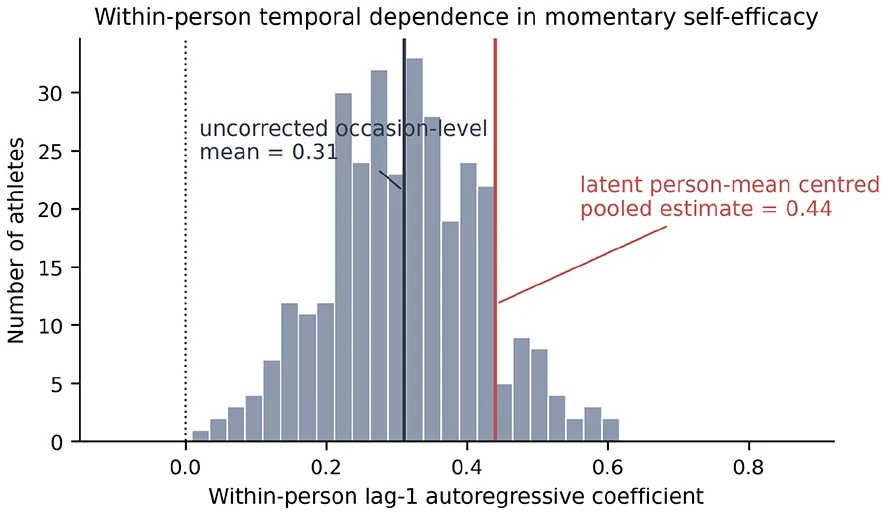
Distribution of individual lag-1 autoregressive coefficients for momentary self-efficacy across the retained athletes, with the pooled estimate obtained under latent person-mean centering and the attenuated estimate obtained without it marked for comparison.

Turning from shape to spread, the distribution of fluctuation amplitude across the sample was strikingly uneven. Figure 10 displays the two indices side by side and both lean right: most adolescents clustered at modest volatility while a sizable tail moved through large, abrupt changes. That upper tail is the subgroup of practical concern, since it is where unstable confidence might accompany erratic training, a possibility the next section takes up directly (Koval et al., 2013).

**Figure 10:**
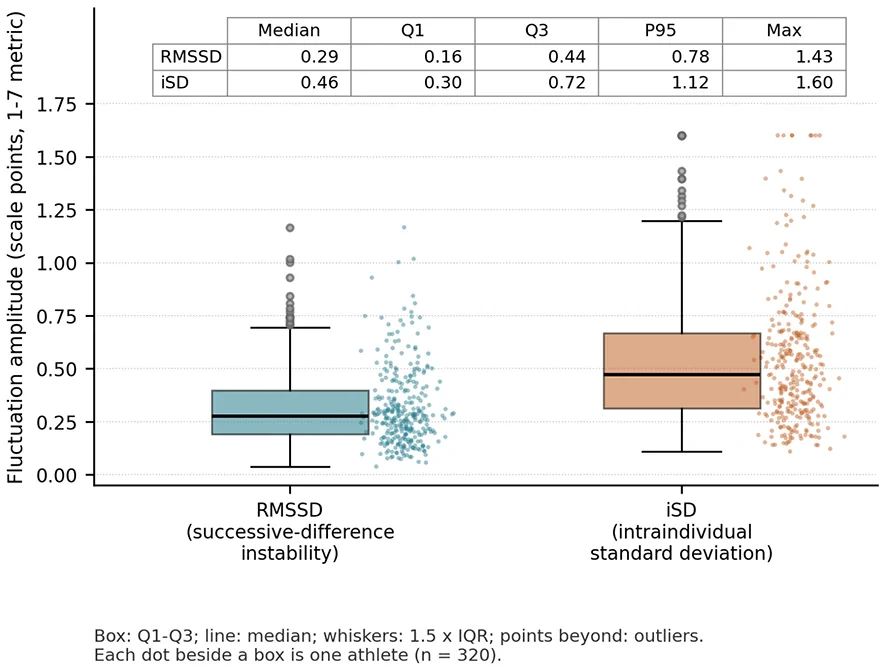
Distribution of the two fluctuation indices across the sample, both expressed in scale points of the seven-point momentary efficacy metric. Boxes span the first to the third quartile, the horizontal line marks the median, and whiskers extend to the most extreme observation lying within 1.5 times the interquartile range of the quartiles; points drawn beyond the whiskers are outliers under that rule. Each dot to the right of a box is one athlete (*n* = 320), jittered horizontally for legibility, and the inset reports the corresponding order statistics. Both distributions lean right, leaving a substantial upper tail of athletes whose confidence moves through pronounced swings.

Together, these patterns answer the descriptive question H4 posed. Efficacy in this population is patterned, temporally dependent, phase-linked, and unevenly distributed, not random scatter around a fixed point. Whether that patterning carries behavioral weight is the question we turn to next.

### 4.2 Testing the effect of online social feedback on efficacy fluctuation

Two distinct questions are addressed in this section, and it is worth separating them before any coefficients are interpreted. The first is an event-level question: when an adolescent encounters an unfavorable comparison, does their confidence move more sharply between that prompt and the next? Its outcome is the absolute occasion-to-occasion change, a within-person quantity that varies from moment to moment. The second is a person-level question: do athletes exposed to more feedback, or to more unfavorable feedback, show wider swings overall? Its outcome is the amplitude index, one value per athlete. The first question is addressed by Equation 9, and the second by Equation 8. The hypotheses map onto them as the lagged and valence claims and as H5 and H7, respectively.

Beginning with the event-level model, the multilevel specification separated feedback by valence, since lumping positive and negative signals together would have masked the asymmetry anticipated in Section 2.1. To probe whether effects were immediate or carried over, each valence entered both contemporaneously and at a one-occasion lag, with the contemporaneous and lagged terms specified in Equation 9:


$$\begin{array}{l} \Delta SE_{i,t}=\gamma_{00}+\gamma_{10}\;Neg_{i,t}+\gamma_{20}\;Neg_{i,t-1}+\gamma_{30}\;Pos_{i,t}\\ \;+\gamma_{40}\;Pos_{i,t-1}+u_{0i}+r_{i,t} \end{array}$$ (9)

in which the outcome is the absolute change in momentary efficacy between adjacent occasions, the event-level indicator of instability defined in Section 3.2, and all occasion-level predictors are person-mean centered (Trull et al., 2008).

Table 7 reports the fixed effects together with the random-effects structure, the latter being indispensable to the cross-level claim that follows. As the results indicate, negatively valenced comparison produced the strongest immediate increase in momentary instability, and its lagged term remained reliable, so the disturbance did not dissipate by the next prompt but carried into the following interval (Ram and Gerstorf, 2009). Positive feedback told a quieter story: its contemporaneous coefficient was smaller, and its lag fell short of conventional thresholds, suggesting that affirming signals briefly boosted confidence without leaving a durable trace. The asymmetry is the most diagnostic feature here, since negative comparison registered more strongly and persisted longer, which fits the broader observation that adolescents weight unfavorable social information disproportionately (Vaish et al., 2008). The random slope for negative feedback showed reliable variance, confirming that reactivity is itself an individual difference rather than a constant, which makes the cross-level interaction meaningful.

**Table 7:** Fixed effects and random-effects structure from the multilevel model predicting event-level momentary instability (Level 1 outcome; comparison orientation is a Level 2 moderator).

Predictor	Estimate	SE	*t*/*z*	*p*
Intercept	0.379	0.030	12.63	<0.001
Negative feedback (concurrent, Level 1)	0.268	0.049	5.47	<0.001
Negative feedback (lag-1, Level 1)	0.141	0.041	3.44	<0.001
Positive feedback (concurrent, Level 1)	−0.107	0.039	−2.74	0.006
Positive feedback (lag-1, Level 1)	−0.038	0.040	−0.95	0.342
Comparison orientation W (Level 2)	0.163	0.051	3.20	0.001
Negative × W (cross-level)	0.187	0.059	3.17	0.002
Positive × W (cross-level)	−0.029	0.049	−0.59	0.555
Posting frequency (Level 1)	0.079	0.030	2.63	0.009
Random intercept variance	0.121	0.018	6.72	<0.001
Random slope variance, negative feedback	0.043	0.011	3.91	<0.001
Intercept–slope covariance	−0.018	0.007	−2.57	0.010
Residual variance	0.226	0.004	56.50	<0.001

Because the lagged coefficient is one of the paper's headline results, we re-estimated it in the dynamic framework described in Section 3.3, where the person mean is treated as latent rather than substituted by the observed average. The lagged association survived that correction and, as expected, was somewhat larger under latent centering (0.163), with a credible interval of [0.081, 0.246], than the 0.141 obtained from the raw multilevel model. The substantive reading is unchanged; the estimator, not the conclusion, was what needed repair (McNeish and Hamaker, 2020).

The moderation pattern further sharpened the picture. The cross-level interaction of comparison orientation with negative feedback was reliable and positive, whereas its interaction with positive feedback was negligible, meaning the disposition amplified vulnerability to unfavorable signals without correspondingly magnifying the benefit of favorable ones (Appel et al., 2016). Figure 11 renders this interaction across the joint range of feedback valence and comparison orientation. As Figure 11 depicts, predicted instability is highest where high negative feedback meets high orientation, while the corresponding positive-feedback surface stays comparatively flat across all orientation levels.

**Figure 11:**
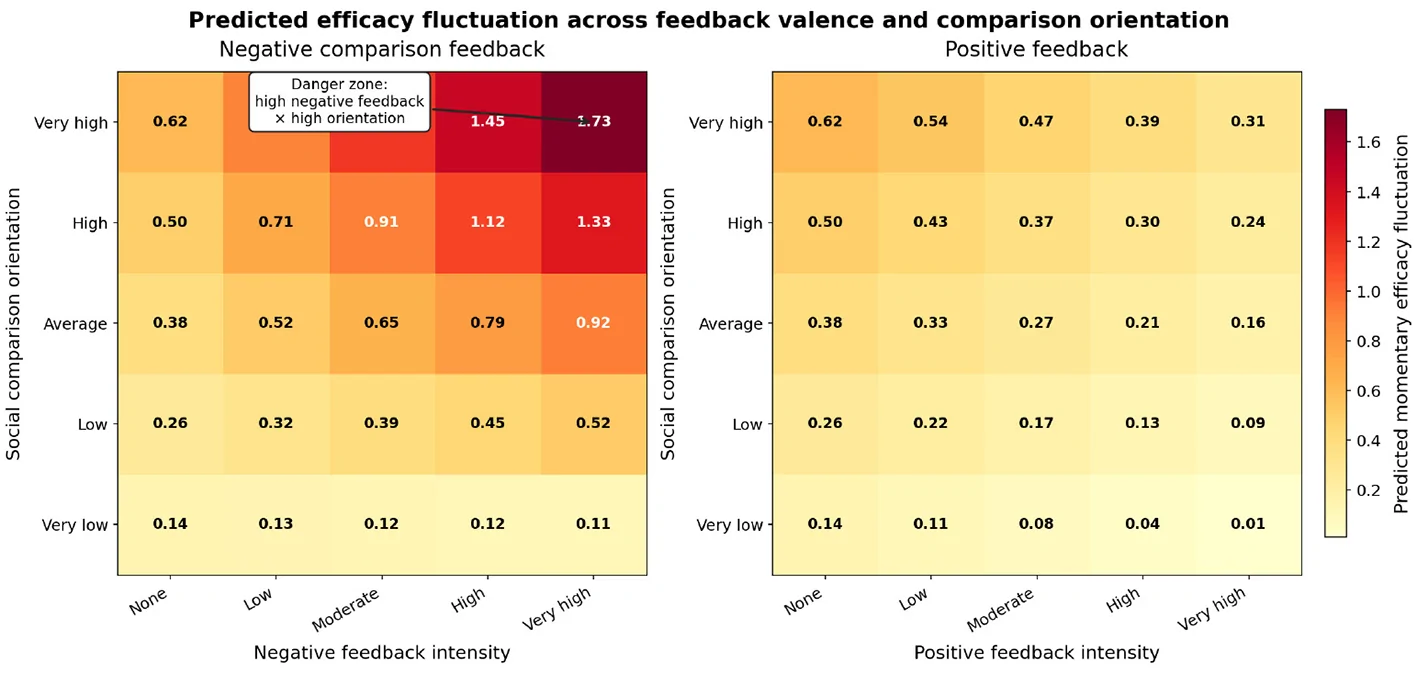
Heatmap of predicted momentary efficacy instability across crossed levels of feedback valence and social comparison orientation, with the most pronounced instability concentrated in the high-negative-feedback, high-orientation quadrant and a markedly flatter surface for positive feedback.

A different model answers the person-level question, and its results qualify the exposure claim in an instructive way. Table 8 regresses each amplitude index on feedback volume, negative comparison intensity, comparison orientation, the two products, and the person mean of momentary efficacy. As Table 8 indicates, H5a receives no clear support: feedback volume was not reliably associated with amplitude once valence was in the model, and the coefficient was small in both specifications. H5b, by contrast, was strongly supported, with negative comparison intensity accounting for the bulk of the explained variance. H7 was likewise supported, since the product of negative intensity and comparison orientation was reliable for both indices. The volume-by-orientation product reached conventional significance for the sequential index alone and not for the dispersion index, and we treat it as unresolved rather than as a finding. The mean efficacy control retained a reliable negative coefficient, consistent with the modest arithmetic entanglement documented in Figure 6 and confirming the value of holding it constant.

**Table 8:** Between-person (Level 2) regressions of fluctuation amplitude on feedback volume, negative comparison intensity and comparison orientation, with the person mean of momentary efficacy controlled.

Predictor	RMSSD: β (SE)	*p*	iSD: β (SE)	*p*
Feedback volume (H5a)	0.076 (0.042)	0.070	0.064 (0.044)	0.143
Negative comparison intensity (H5b)	0.480 (0.043)	<0.001	0.451 (0.045)	<0.001
Comparison orientation W	0.417 (0.036)	<0.001	0.410 (0.038)	<0.001
Volume × W	−0.086 (0.042)	0.041	−0.042 (0.044)	0.334
Negative intensity × W (H7)	0.313 (0.039)	<0.001	0.278 (0.041)	<0.001
Person mean efficacy (control)	−0.138 (0.035)	<0.001	−0.218 (0.037)	<0.001
Model *R*^2^	0.624	–	0.588	–

All variables are standardized.

Taken together, the two models refine rather than simply confirm the original claims. Momentary instability rises with unfavorable comparison, immediately and with a lag, and athletes disposed to compare display wider amplitude overall. The data do not support the proposition that exposure as such, understood as the volume of evaluative feedback, increases fluctuation; rather, the tone of the feedback, rather than its density, carries the association. The practically important result remains the persistence of the lagged effect: a single bruising comparison does not simply register and clear, and the cost of exposure appears to accumulate across intervals rather than resetting at each occasion (Kross et al., 2013). That reading concerns a within-person process and rests on the within-person estimates in Table 7, not on the between-person associations in Table 8. We can address two rival readings in part rather than defer them. Because the competitive calendar loads unfavorable comparison and training stress onto the same weeks, we re-estimated the lagged model with calendar phase as a time-varying covariate and then within each phase separately; and because competitive tier might govern platform habits and the meaning of engagement alike, we entered it as a Level-2 covariate and tested its interaction with negative comparison. Neither disturbed the conclusions, and we report both with the other supplementary analyses in Table 11.

### 4.3 Testing the influence pathway from efficacy fluctuation to training engagement

The final structural model brought the social, psychological, and behavioral layers into one estimable system, letting us ask whether the fluctuation documented above accompanies training engagement and, if so, along which route. Before reading any coefficient, we checked global fit, since a path table from a misfitting model would be worse than no table at all. The model fit the data well, with the ratio of chi-square to degrees of freedom comfortably below the conventional ceiling and the fit indices reported in Equation 10:


$$\begin{array}{l} RMSEA=0.041,\;CFI=0.962,\;TLI=0.951,\;SRMR=0.048 \end{array}$$ (10)

These indices all met the thresholds usually demanded for adequate-to-good fit (Hu and Bentler, 1999). We estimated the model as a multilevel structural equation model with an explicit within-between decomposition, so the occasion-level antecedent, the person-level mediator, and the person-level outcomes each enter at their own level (Preacher et al., 2010). With that assurance, we turned to decomposing the effects.

Table 9 presents the path coefficients alongside the mediation decomposition. As Table 9 shows, fluctuation amplitude had a reliable negative association with all three engagement facets, and the magnitudes varied: absorption showed the largest coefficient, vigor an intermediate one, and dedication the smallest. The direct effects of negative comparison are reported facet by facet rather than on a composite, as H1 requires: the association was reliable for absorption and vigor and did not reach conventional significance for dedication, which is itself informative about where the residual pathway operates. The indirect path from negative comparison through amplitude to engagement was reliable, with the Monte Carlo interval for the mediated portion excluding zero, while the residual direct effect shrank without vanishing, marking partial rather than full mediation. Because significance alone says little about magnitude, the lower rows report a standardized mediation effect size of 0.09, which places the mediated portion in the small-to-moderate range and is the figure we would ask a reader to carry away (Preacher and Kelley, 2011). The proportion of the total effect carried indirectly, 0.46, appears beside it for completeness, but it should be read with more caution than its apparent precision suggests: in partial-mediation models this ratio is an unstable statistic with a wide sampling distribution, as the simulation literature established some time ago (MacKinnon et al., 1995), and the interval obtained here, running from 0.30 to 0.64, shows the same width. The practical claim of this study rests on the standardized effect, not on the arithmetic that turns it into a percentage.

**Table 9:** Path coefficients and decomposition of direct, indirect, and total effects on training engagement, with levels indicated.

Path/effect	β	SE	95% CI	Conclusion
Negative feedback (L1) → fluctuation amplitude (L2)	0.341	0.051	[0.241, 0.441]	Supported
Amplitude (L2) → vigor (L2)	−0.291	0.061	[−0.411, −0.171]	Supported
Amplitude (L2) → dedication (L2)	−0.183	0.051	[−0.283, −0.083]	Supported
Amplitude (L2) → absorption (L2)	−0.372	0.061	[−0.492, −0.252]	Supported
Mean efficacy (L2) → engagement (L2)	0.312	0.051	[0.212, 0.412]	Supported
Direct: negative feedback → vigor	−0.112	0.051	[−0.212, −0.012]	Supported
Direct: negative feedback → dedication	−0.089	0.051	[−0.189, 0.011]	Not supported
Direct: negative feedback → absorption	−0.158	0.051	[−0.258, −0.058]	Supported
Indirect via amplitude (composite outcome)	−0.112	0.031	[−0.182, −0.052]	Supported
Total effect (composite outcome)	−0.241	0.061	[−0.361, −0.121]	Supported
Proportion of total effect mediated	0.464	0.087	[0.301, 0.638]	–
Standardized mediation effect size	0.091	0.028	[0.041, 0.152]	–
Index of moderated mediation, H3 (mean path, negative arm)	−0.047	0.021	[−0.091, −0.009]	Supported
Index of moderated mediation, H3 (mean path, positive arm)	0.011	0.019	[−0.027, 0.048]	Not supported
Index of moderated mediation, H7 (amplitude path)	−0.071	0.031	[−0.133, −0.019]	Supported
Mean efficacy (control on amplitude)	−0.152	0.051	[−0.252, −0.052]	–

Fluctuation amplitude is operationalized throughout this model by the root mean square of successive differences alone; the parallel model using the intraindividual standard deviation is reported among the robustness checks in Table 10. Figure 13 decomposes the composite indirect effect facet by facet.

The contrast in the middle rows is the substantive core of H6: mean efficacy retained its positive association with engagement even as amplitude carried a separate negative one, which addresses the worry that instability might be nothing more than disguised low confidence (Preacher et al., 2007). The two operate apart, so that an athlete can hold a healthy average belief and still show reduced engagement in the presence of volatility. The two indices of moderated mediation, reported separately for the mean path and the amplitude path as H3 and H7, respectively, both excluded zero on the negative arm but not the positive arm, so the conditional pattern is valence-specific and cannot be represented by a single pooled index.

The claim that fluctuation strikes hardest at absorption needed a formal test rather than a visual comparison of coefficients, and Table 10 supplies it. Constraining the three amplitude-to-facet paths to equality worsened fit relative to the freely estimated model, so the facets are not interchangeable, as the control proposition in Table 1 anticipated. The pairwise comparisons, however, license a narrower conclusion than the ordering of the coefficients alone would suggest: absorption differed reliably from dedication, but absorption and vigor could not be distinguished, and neither could vigor and dedication. The defensible statement is therefore that the association is graded across the facets with absorption at one end and dedication at the other, not that absorption is singularly affected. Figure 12 makes the overlap visible.

**Table 10:** Nested model comparisons and supplementary tests bearing on facet specificity, directionality and index choice.

Test	Statistic	*p*	Interpretation
Three amplitude-to-facet paths constrained equal vs. freely estimated	Δχ^2^_(2)_ = 6.81	0.033	Facets not interchangeable
Absorption vs. dedication	χ^2^_(1)_ = 5.92	0.015	Reliably different
Absorption vs. vigor	χ^2^_(1)_ = 0.89	0.346	Not distinguishable
Vigor vs. dedication	χ^2^_(1)_ = 1.98	0.159	Not distinguishable
Reverse within-person path: efficacy → later comparison browsing	*b* = −0.004 (0.010)	0.668	No reciprocal evidence
Forward within-person path under latent centring	*b* = 0.163 (0.042)	<0.001	Directional reading supported
Structural model re-estimated with the dispersion index	max |Δβ| = 0.028	–	Conclusions unchanged
Structural model with baseline trait engagement added	max |Δβ| = 0.021	–	Conclusions unchanged

**Figure 12:**
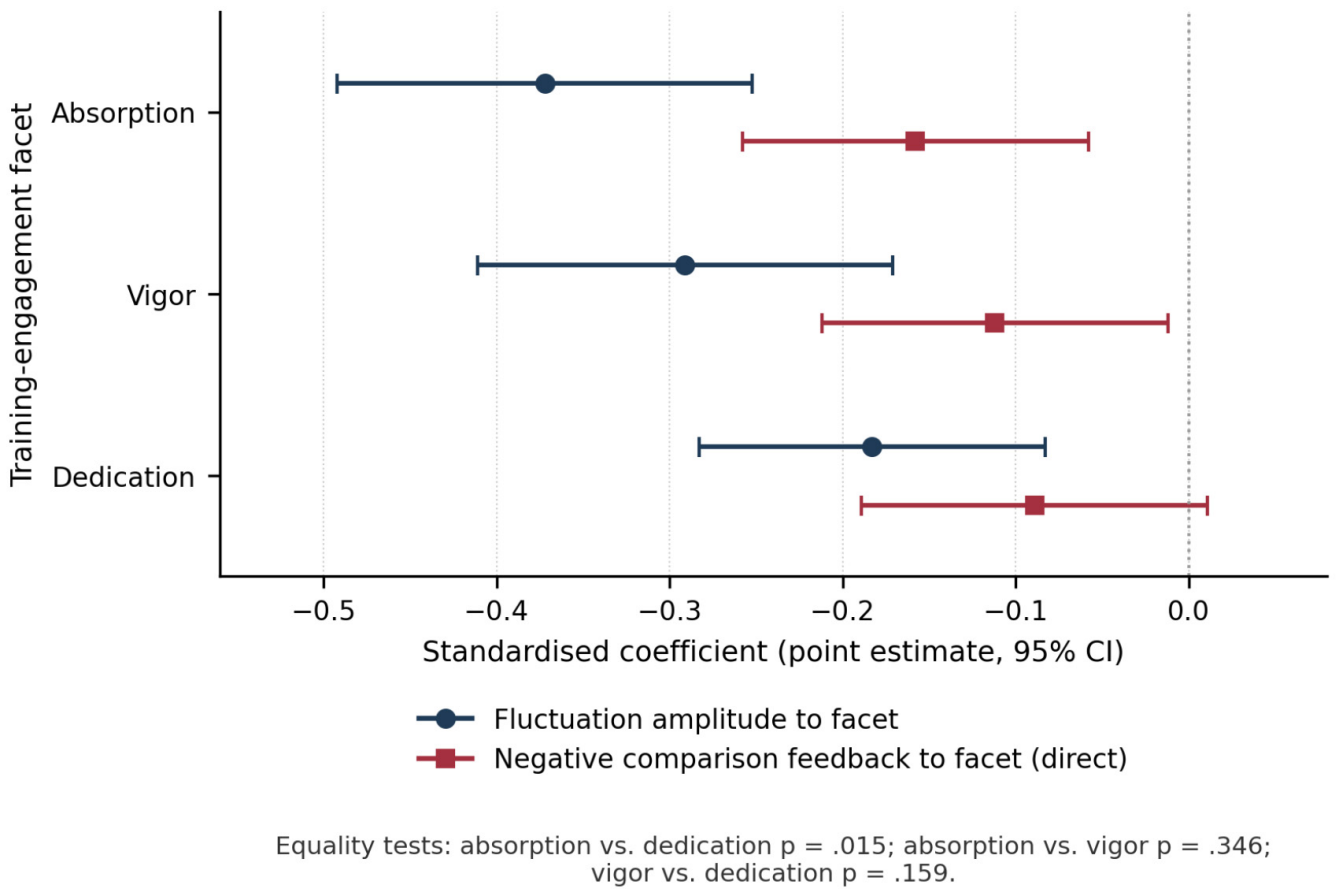
Facet-specific standardized coefficients for the association between fluctuation amplitude and each engagement facet, shown together with the direct effects of negative comparison feedback on the same facets. Horizontal bars are 95% confidence intervals around the point estimate, and the vertical dotted line marks zero. All three equality tests on the amplitude paths are reported beneath the panel, so that the single reliable contrast can be read against the two that are not; the overlap of absorption with vigor is visible directly.

Figure 13 compares the standardized indirect effects across the three facets. As it shows, the mediated portion is largest for absorption and smallest for dedication, a gradient consistent with the conceptual ordering set out in Section 2.2, in which absorption draws on present-moment attentional resources while dedication rests on a slower-forming identity investment.

**Figure 13:**
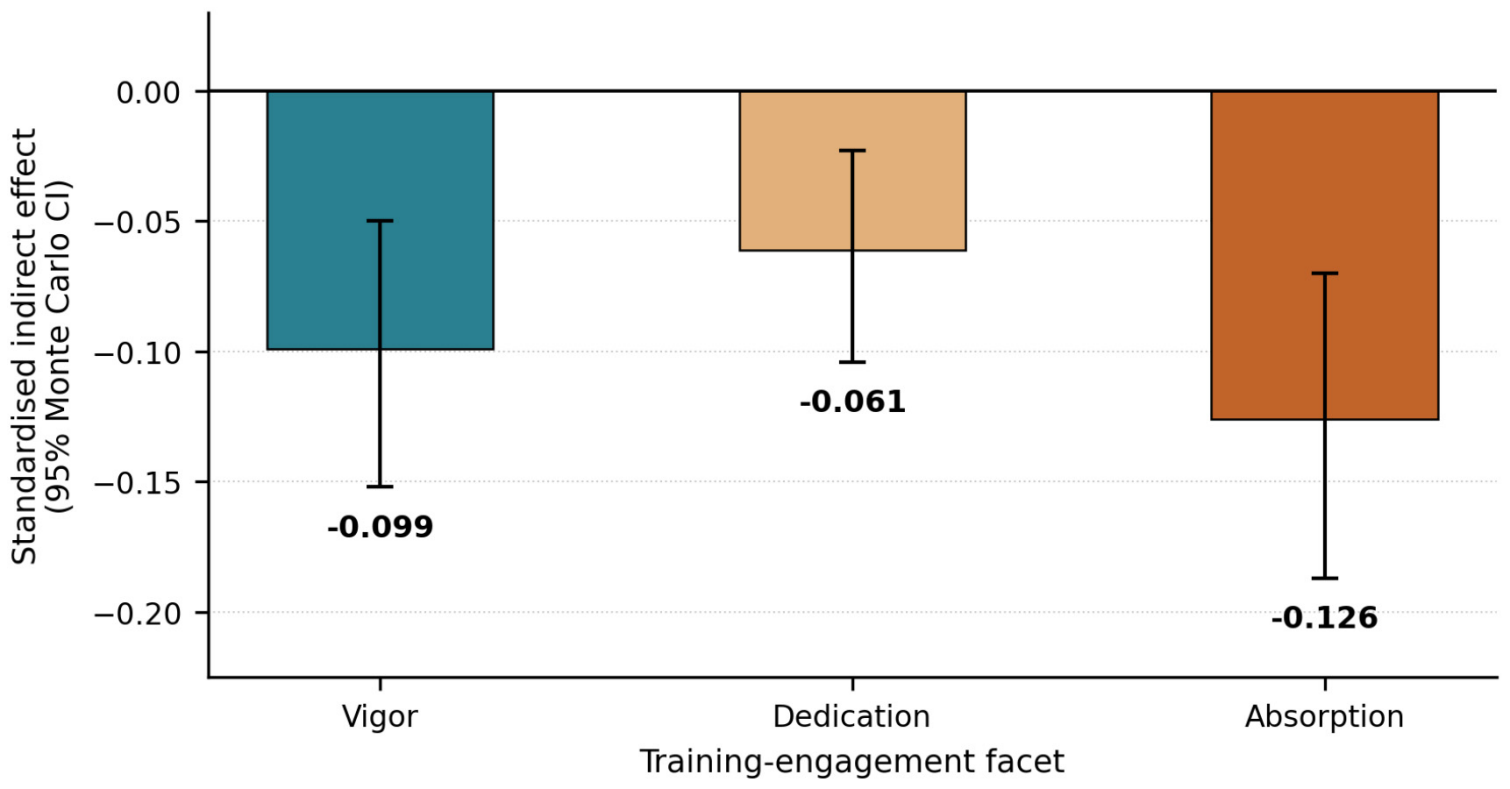
Standardized indirect effects of online negative comparison feedback on the three training-engagement facets, transmitted through self-efficacy fluctuation amplitude. Vertical bars are 95% Monte Carlo confidence intervals, and the plotted value is printed beneath each bar. The mediated reduction is largest for absorption, intermediate for vigor, and smallest for dedication; the corresponding total and direct effects appear in Figure 12 and Table 9, which are not repeated here.

Robustness checks left these conclusions standing, and the lower rows of Table 10 report them. Re-estimating the structural model with the dispersion index in place of the sequential one preserved the sign and rough magnitude of every path, which is unsurprising given that the two indices correlate at 0.88 in this sample; adding baseline trait engagement as a covariate did not overturn the mediation. One test did not return a null of the convenient kind. The reverse within-person path, from momentary efficacy to subsequent comparison-relevant browsing, was estimated because a self-perpetuating account requires the traffic run in the stated direction, not merely that the two covary (Hamaker et al., 2015). Its coefficient was indistinguishable from zero, which is reassuring for the directional reading but should not be over-interpreted, since a null in a single reverse path is weaker evidence than a positive finding in the forward one.

Four further specifications, shown in Table 11, address the rival readings that an observational design invites. Adding calendar phase as a time-varying covariate left the lagged effect of negative comparison almost exactly where it had been, and splitting the series by phase returned three estimates differing by less than their standard errors, so the lagged disturbance is not an artifact of the weeks in which load and fixtures pile up; the phase indicator did carry a modest effect of its own, which is what one would expect if load contributes something without accounting for the association. Competitive tier neither predicted amplitude nor moderated the effect of negative comparison, though the national subgroup is small and the test correspondingly weak. Substituting the residualized amplitude score, in which the person mean and the person-specific autoregression have been partialled out, attenuated the amplitude-to-facet paths by roughly a 10th of their size and left every one of them reliable—an outcome the skeptical reading of a volatility index does not predict, since on that reading the residual should have carried nothing. Automated sentiment polarity agreed substantially with participant-coded valence on the free-text subset, and, more to the point, that agreement did not vary with comparison orientation, so the cross-level interaction of Table 7 is unlikely to be a coding artifact. As Table 11 shows, none of the four specifications overturns a reported result; Figure 14 sets the phase-split estimates and the residualized paths side by side.

**Table 11:** Supplementary sensitivity analyses bearing on calendar-phase confounding, competitive tier, the residualized amplitude index and the validity of participant-coded valence.

Supplementary specification	Estimate	*p*	Interpretation
Lagged model with calendar phase added: negative comparison, lag-1 (Level 1)	*b* = 0.132 (0.042)	0.002	Lagged effect retained
Calendar phase indicator, high-load block vs. ordinary weeks (Level 1)	*b* = 0.061 (0.026)	0.019	Small independent increment
Split sample, high-load block: negative comparison, lag-1	*b* = 0.148 (0.055)	0.007	Stable across phases
Split sample, competition weeks: negative comparison, lag-1	*b* = 0.126 (0.061)	0.039	Stable across phases
Split sample, post-competition weeks: negative comparison, lag-1	*b* = 0.119 (0.058)	0.041	Stable across phases
Competitive tier entered as a Level-2 covariate of amplitude	*b* = −0.032 (0.041)	0.434	No tier effect
Competitive tier × negative comparison intensity (Level 2)	Δχ^2^_(2)_ = 2.14	0.343	No moderation by tier
Residualised amplitude → vigor (mean and autoregression partialled)	β = −0.254 (0.063)	<0.001	Effect retained
Residualised amplitude → dedication	β = −0.151 (0.053)	0.004	Effect retained
Residualised amplitude → absorption	β = −0.331 (0.062)	<0.001	Effect retained
Indirect effect via residualized amplitude (composite outcome)	β = −0.096 (0.029)	0.001	Mediation retained
Automated sentiment polarity vs. participant-coded valence (free-text subset)	κ = 0.71	–	Substantial agreement
Comparison orientation × sentiment–participant agreement	*b* = −0.028 (0.031)	0.368	No coding-bias evidence

Standard errors appear in parentheses; b denotes an unstandardized coefficient and β a standardized coefficient.

**Figure 14:**
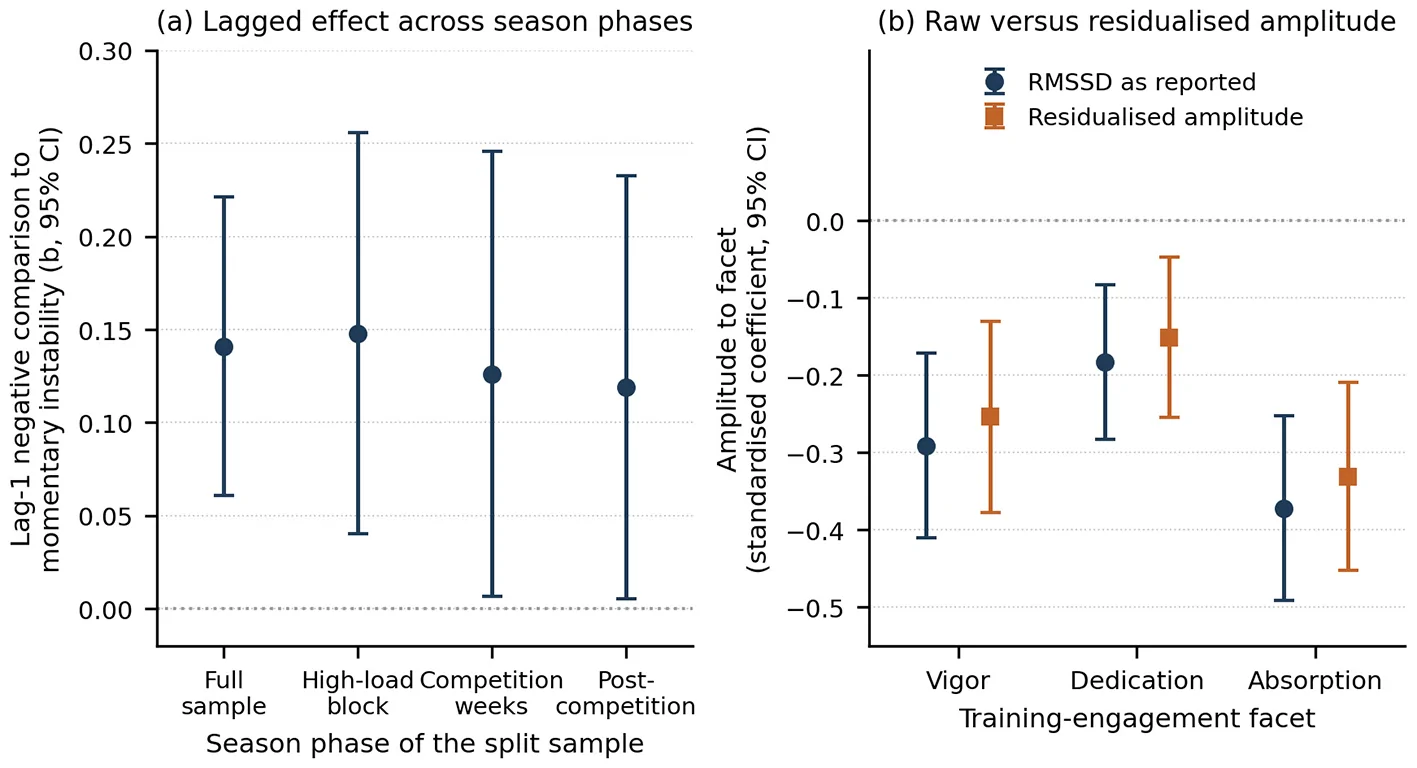
Sensitivity of the two headline estimates. Panel **(a)** plots the lag-1 effect of negative comparison on momentary instability in the full sample and within each season-phase subsample, with 95% confidence intervals; panel **(b)** contrasts the standardized amplitude-to-facet coefficients obtained with the sequential index as reported and with the residualized amplitude score, in which the person mean and the person-specific lag-1 autoregressive parameter have been partialled out.

Read across Sections 4.1 to 4.3, the evidence supports H2, H4, H5b, H6, and H7. It does not support H5a, the volume-based half of the exposure claim, and it supports H1 only in part: negative comparison depressed vigor and absorption directly but left dedication where it found it. H3 holds on the negative arm of feedback, not the positive one, and the facet pattern is graded rather than singular. Stating the verdicts this way, rather than reporting broad support, is what the coefficients actually license.

## 5 Discussion

This study clarifies that the movement of confidence, not merely its level, is associated with the behavioral outcome of interest. Before drawing that thread out, it is worth marking which evidence sits at which level, because the two do not license the same inferences and a within-person association need not agree in sign or direction with a between-person one. The temporal dependence in efficacy, the immediate and lagged response to unfavorable comparison, and the absence of a reverse path are all within-person findings estimated inside individual series. The associations between amplitude and engagement, the moderating role of comparison orientation on amplitude, and the negative relation between exposure profile and trajectory slope are all between-person findings that describe how athletes differ from one another. Statements about what happens to an athlete after a bruising comparison rest on the first set; statements about which athletes train less well rest on the second. Section IV focuses on estimation and reporting; the interpretive weight is carried here. One reading follows immediately from the moderation pattern reported there. Athletes high in comparison orientation pay the cost of an unfavorable feed twice over, once through a higher baseline level of instability and again through a steeper reaction to each negative event, while gaining no corresponding extra protection from praise (Appel et al., 2016).

The lagged effect of negative comparison, a within-person result, is consistent with a self-perpetuating process in which an unfavorable signal widens the present swing and the widened swing carries into the next interval, so that an adolescent in a comparison-dense feed may settle into a regime of instability rather than returning to a flat baseline. The design is observational, so the language here is deliberately associational: temporal precedence and the null reverse path make the directional reading more plausible than its converse, but neither establishes causation, and an unmeasured common cause remains possible. The coupling with the training calendar deepens the picture without changing its status, since competitive nodes and post-event posting peaks coincided, meaning the moments of highest stakes are also when athletes expose themselves most to the signals associated with instability. That coupling is also this study's clearest alternative explanation, and it deserves naming rather than smoothing over. A competition week is simultaneously the peak of unfavorable comparison and the peak of accumulated load, fatigue, and result-related stress, and either could move efficacy and engagement on its own (Impellizzeri et al., 2019). The sensitivity analyses of Table 11 narrow the gap without closing it. The lagged effect survives the addition of calendar phase and holds within each phase, which sits awkwardly with a purely load-driven account; yet calendar phase is a coarse proxy, and no session ratings, wearable measures, placings, or recovery scores were collected. Until such indicators travel alongside the momentary reports, the associational language used here is a necessity rather than a display of modesty.

Set against existing work, the contribution reframes the vicarious and persuasive sources of social cognitive theory for a digital ecology. Where the classical account treated these sources as discrete, occasional events feeding a slowly built belief, the present findings show them arriving as a quantified, asymmetric stream that leaves a measurable dynamic signature. Negative weighting dominates, positive signals fade quickly, and dispositional comparison tendency tunes the gain on the negative channel alone. This extends the theory into a temporal regime the original formulation never had to address rather than refuting it, and it does so with the tempering supplied by recent meta-analytic work: the confidence–performance relation in sport is modest and moderated (Lochbaum et al., 2022), so claims about what stabilizing confidence would buy in training terms should stay correspondingly measured.

The transience of the positive effect deserves more than a passing note, because it is the result most likely to surprise a practitioner. Two accounts, both compatible with the data, suggest themselves. The first is an expectancy account: adolescents embedded in platform culture receive affirming signals continuously, so a like or a supportive comment largely confirms what was already anticipated and, carrying little prediction error, prompts little belief revision. The second is a diagnosticity account drawn from cognitive appraisal and affective information-processing frameworks: negative information is treated as more informative about capability than positive information of equal magnitude, because a failure signal constrains the range of plausible self-assessments more sharply than a success signal does. On this reading, praise is processed as ambient and criticism as evidence, and the asymmetry in persistence follows directly. The present design cannot adjudicate between the two, since it did not measure expectancy or perceived diagnosticity, but the distinction is testable and worth building into future protocols.

Symmetry demands the converse question, and it is a question easily left unasked: if praise fades, why does criticism linger into the following interval? The most economical answer is that an unfavorable comparison does not end when the screen goes dark. Repetitive self-focused thought keeps the discrepancy active, rehearses it, and thereby holds the belief system in a disturbed state well past the event that disturbed it—the process the affective literature calls rumination and documents as a principal reason negative material outlasts its trigger (Nolen-Hoeksema et al., 2008). Read as cognitive fixation, this account maps closely onto Table 7: the contemporaneous coefficient marks the arrival of the signal, the lagged coefficient marks the fact that attention has not yet released it, and the missing positive lag follows because affirming signals seldom recruit rehearsal at all. The proposal is a hypothesis rather than a finding, since rumination went unmeasured; but it is cheaply testable: a two-item momentary probe of intrusive replay would suffice, and it ties the lag documented here to the absorption gradient of Section 4.3, since rehearsal competes for exactly the attentional resources on which absorption depends.

The practical implications follow from the asymmetry and from the lag, and they can be stated more concretely than a general call for awareness. Because the disturbance associated with an unfavorable comparison was still detectable at the following prompt, a plausible point of intervention is the interval itself. Coaches might treat the window immediately after competition, when posting and comparison both peak, as a period requiring active rather than passive support: a brief structured efficacy-reflection conversation within 24 h of the event, organized around what the athlete controlled, what the result does and does not indicate about capability, and what the next session will target, is a low-cost way of supplying an authoritative interpretation before the feed supplies one. For athletes themselves, recognizing that a bruising comparison is likely to color the next training session, rather than being a self-contained episode, offers a foothold for self-regulation that targeting average confidence would miss; a simple monitoring habit, noting confidence before and after feed use in the post-competition period, makes the pattern visible without requiring instrumentation. Curating comparison targets and spacing feed engagement away from high-load blocks are likewise more workable than blanket restriction. These recommendations bear on within-person processes and are grounded in the lagged estimates of Table 7; the between-person findings, by contrast, support a screening use, identifying athletes whose disposition and exposure profile place them in the high-amplitude tail, rather than a timing use. None of this presumes that the association is causal. Each recommendation is inexpensive and defensible on its own terms, which is the appropriate standard for advice drawn from an observational design.

## 6 Conclusion

This study set out to read adolescent athletes' self-efficacy not as a fixed quantity but as a process in motion, and the effort paid off in several connected findings. Confidence over the 8-week window proved temporally dependent rather than random, phase-linked to the training calendar and unevenly spread across individuals, with a worrying tail of athletes whose beliefs moved sharply between adjacent occasions. Online social feedback was associated with that volatility asymmetrically: negative comparison enlarged momentary instability both at once and into the following interval, positive signals steadied confidence only briefly, and a comparison-prone disposition sharpened sensitivity to the negative arm alone. Between persons, the intensity of unfavorable comparison rather than the volume of feedback tracked amplitude. The fluctuation so indexed was in turn negatively related to training engagement through a partial-mediation route whose standardized effect size was small to moderate, strongest for in-task absorption and least for dedication, though a formal test separated absorption from dedication only. Negative comparison also reached vigor and absorption directly while leaving dedication untouched, so the facet-by-facet form of the first hypothesis is supported in part rather than in full, and the volume-based half of the exposure hypothesis is not supported at all.

The contribution sits at two levels. Methodologically, treating efficacy as a dynamic signal, indexed by within-person dispersion and successive-difference instability, and estimating it with the within- and between-person components kept apart, recovers information that snapshot designs discard. Theoretically, joining the social, psychological, and behavioral layers into one estimable path extends social cognitive theory's account of vicarious and persuasive sources into a digital, temporally dense regime it was never built to describe.

Several limitations bound these conclusions, and one of them outranks the rest. No objective indicator of training load, competition outcome, or recovery status was collected, so the models cannot separate the psychological consequences of unfavorable comparison from the physiological and competitive stress that arrives in the same weeks. Calendar phase, entered as a time-varying covariate and used to split the sample, is the best proxy the design affords, and the estimates survived it; a proxy is nevertheless not a measurement, and a study fusing momentary self-report with session ratings of perceived exertion, wearable load and recovery monitoring would settle what this one can only bound. Three further limitations qualify the reading. The sample, drawn from one cultural and competitive setting, limits generalization, and its national-tier subgroup is small enough that the tier analyses reported in Table 11 should be read as exploratory rather than conclusive. The valence of each feedback event was coded by a single forced-choice item completed by the participant, so the interaction between comparison orientation and negative comparison could in principle reflect differences in coding threshold as much as differences in reactivity; the sentiment check reported in Section 4.3 speaks against that reading without eliminating it. Signal-contingent sampling within fixed windows, finally, captures ordinary days well but may miss the most charged moments, and 8 weeks captures only a slice of an athletic season. Two long-standing concerns remain in force. The first is common-method variance: momentary efficacy, feedback, engagement, and comparison orientation were all self-reported, so shared method effects cannot be excluded. Temporal separation between the momentary and weekly instruments mitigates this for the paths linking occasion-level feedback to weekly engagement, but it does not help for associations estimated within a single prompt, and those coefficients should be read with corresponding caution. The second is causal identification. This is an observational design, and every inference about mediation direction rests on theoretical assumption and model specification rather than on manipulation. Temporal precedence provides a clue, and the null reverse path removes one rival reading, but neither rules out unmeasured third variables, most obviously competition results themselves, coaching climate, sleep and physical condition, or menstrual-cycle and growth-related fluctuations, any of which could move efficacy and engagement together and generate the observed association without any path running between them.

Future work follows fairly directly from those limits. Fusing self-report with passive behavioral and physiological streams would loosen the dependence on a single method, and adding event-contingent prompts, triggered by a post, a bout of comparison-relevant browsing, or the end of a competition, would catch the moments that a fixed window can only sample by luck (Bolger and Laurenceau, 2013). A brief momentary measure of rumination would convert the persistence account offered above from an interpretation into a testable mechanism, and scoring the text of feedback automatically rather than relying on a single self-coded item would put the valence construct on firmer ground. Testing whether the negativity asymmetry holds across cultures would establish how far the pattern travels. Most importantly, the causal questions call for designs this study could not supply: ecologically valid experimental interventions in which exposure to positive vs. negative comparison content is randomized within participants, and cross-lagged panel network analyses that model the full set of reciprocal relations among feedback, efficacy dynamics and engagement rather than a single hypothesized chain (Hamaker et al., 2015). Whether guided social-media routines actually narrow the swings this study could only chart remains an open and testable question.

## Data Availability

The original contributions presented in the study are included in the article/Supplementary material, further inquiries can be directed to the corresponding author/s.

## Supplementary material

Supplementary Data Sheet 1 De-identified study data, analysis scripts and complete unrounded model output supporting the analyses reported in this article; package versions and the random seed are included with the analysis materials.
